# Integration of Mass Spectrometry Imaging and Machine Learning Visualizes Region-Specific Age-Induced and Drug-Target Metabolic Perturbations in the Brain

**DOI:** 10.1021/acschemneuro.1c00103

**Published:** 2021-05-03

**Authors:** Theodosia Vallianatou, Reza Shariatgorji, Anna Nilsson, Maria Karlgren, Heather Hulme, Elva Fridjonsdottir, Per Svenningsson, Per E. Andrén

**Affiliations:** † Medical Mass Spectrometry Imaging, Department of Pharmaceutical Biosciences, Biomedical Centre 591, Uppsala University, SE-75124 Uppsala, Sweden; ‡ Science for Life Laboratory, Spatial Mass Spectrometry, Biomedical Centre 591, Uppsala University, SE-75124 Uppsala, Sweden; § Department of Pharmacy, Uppsala Drug Optimization and Pharmaceutical Profiling (UDOPP), Biomedical Centre 580, Uppsala University, SE-75123 Uppsala, Sweden; ⊥ Section of Neurology, Department of Clinical Neuroscience, Karolinska Institutet, SE-17177 Stockholm, Sweden

**Keywords:** Acetylcholine, aging, lipids, mass spectrometry imaging, brain metabolomics, tacrine

## Abstract

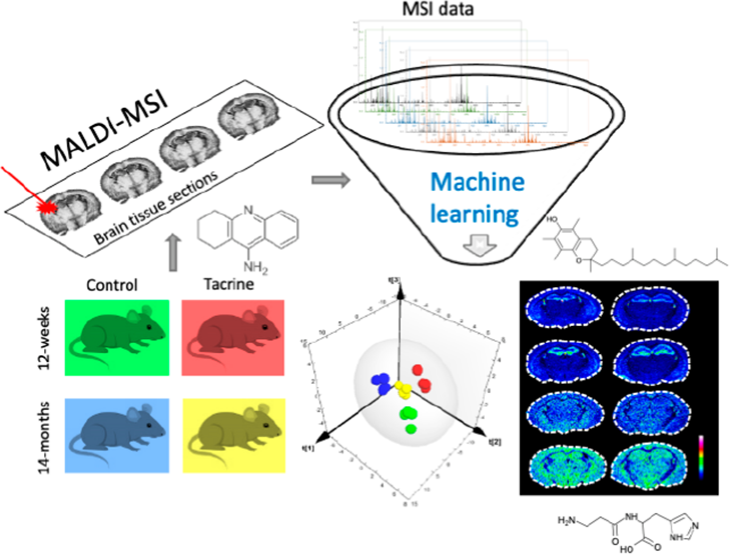

Detailed metabolic imaging of specific brain regions in early aging may expose pathophysiological mechanisms and indicate effective neuropharmacological targets in the onset of cognitive decline. Comprehensive imaging of brain aging and drug-target effects is restricted using conventional methodology. We simultaneously visualized multiple metabolic alterations induced by normal aging in specific regions of mouse brains by integrating Fourier-transform ion cyclotron resonance mass spectrometry imaging and combined supervised and unsupervised machine learning models. We examined the interplay between aging and the response to tacrine-induced acetylcholinesterase inhibition, a well-characterized therapeutic treatment against dementia. The dipeptide carnosine (β-alanyl-l-histidine) and the vitamin α-tocopherol were significantly elevated by aging in different brain regions. l-Carnitine and acetylcholine metabolism were found to be major pathways affected by aging and tacrine administration in a brain region-specific manner, indicating altered mitochondrial function and neurotransmission. The highly interconnected hippocampus and retrosplenial cortex displayed different age-induced alterations in lipids and acylcarnitines, reflecting diverse region-specific metabolic effects. The subregional differences observed in the hippocampal formation of several lipid metabolites demonstrate the unique potential of the technique compared to standard mass spectrometry approaches. An age-induced increase of endogenous antioxidants, such as α-tocopherol, in the hippocampus was detected, suggesting an augmentation of neuroprotective mechanisms in early aging. Our comprehensive imaging approach visualized heterogeneous age-induced metabolic perturbations in mitochondrial function, neurotransmission, and lipid signaling, not always attenuated by acetylcholinesterase inhibition.

## Introduction

Aging constitutes a major risk factor for several neurodegenerative disorders such as Alzheimer’s disease (AD) and Parkinson’s disease (PD).^1^ Owing to the multifactorial nature of aging processes, metabolic profiling of normal brain aging can serve as a valuable tool for better understanding cellular senescence and providing information about the pathophysiology.^2,3^ Untargeted metabolomics studies using mass spectrometry have been widely applied for the identification and quantification of endogenous small molecules affected by aging and have revealed several metabolic pathways mainly related to mitochondrial activity, lipid metabolism, and oxidative stress.^2−6^ However, this technology is limited in providing detailed brain localization information, which is essential for understanding neuronal senescence.

It has been demonstrated that the extent of neurochemical alterations induced by aging differs among and within the various brain regions^4^ as well as between different cell types, such as glia and neurons.^7^ Age-induced degeneration in the hippocampus (Hip) and frontal cortex is related to cognitive decline, while the striatum (Str) is widely associated with age-triggered motor deficiencies.^8,9^ Moreover, other brain regions such as the retrosplenial cortex (RS), a small postcingulate cortical area which is involved in spatial memory and navigation,^10^ have been reported to be one of the first brain regions affected in AD.^11^ In addition, white matter has been shown to play a key role in the aging brain, as myelin breakdown during normal aging has been reported.^12^ Hence, comparative analysis of different brain regions can help to decipher structure-specific neurochemical alterations induced by aging. Most metabolic profiling studies to date only examined the factor of aging. Concomitant investigation of other factors, such as drug central nervous system (CNS) effects, can provide further insights into the responsivity of the aged brain and facilitate in the search for efficacious neuroactive agents.^13,14^

In the present study, we applied ultrahigh mass resolution Fourier-transform ion cyclotron resonance (FTICR) matrix-assisted laser desorption/ionization mass spectrometry imaging (MALDI-MSI) combined with supervised and unsupervised multivariate analysis (MVA) machine learning models to investigate region-specific metabolic differences between 12-week old (adult) and 14-month old (middle age) mice with and without the presence of the acetylcholinesterase (AChE) inhibitor tacrine. We have previously demonstrated that tacrine, a well-established AChE inhibitor, induces regional and age-dependent elevations of acetylcholine levels in the brain.^14^ This neuroactive agent can therefore serve as a model drug for evaluating the age-specific responsivity of multiple neurochemical pathways revealing potential interplays with the cholinergic system. The MALDI-MSI technology enables detailed imaging of small anatomical brain regions and subregions and the application of several machine learning algorithms allows deep and validated examination of the data. We explored the correlation between essential molecular properties of a series of analog metabolites and their regional, age-dependent, as well as drug-related alterations. Finally, we implemented *in vitro* transport experiments to assess specific tacrine effects. Our imaging approach revealed region-specific alterations in the acetylcholine and l-carnitine pathways, brain lipids, and endogenous antioxidants, and it provided numerous molecular images of a tissue section simultaneously, offering unique visualization of biological data.

## Results

Mouse brain tissue sections from two different coronal levels were analyzed by MALDI-MSI, including brain structures such as corpus callosum (cc), cortex (Cx), medial forebrain bundle (mfb), and Str (0.26 mm from bregma) as well as Hip and RS (−1.06 mm from bregma).^15^ The imaging analysis included four groups of mice (*n* = 4 per group): 12-week (12-w control) and 14-month (14-m control) saline-injected mice and 12-week (12-w tacrine) and 14-month (14-m tacrine) tacrine-administered animals. The MALDI-MSI results were further analyzed by MVA.

### Multivariate Imaging Analysis and Metabolite Identification

**Figure 1:**
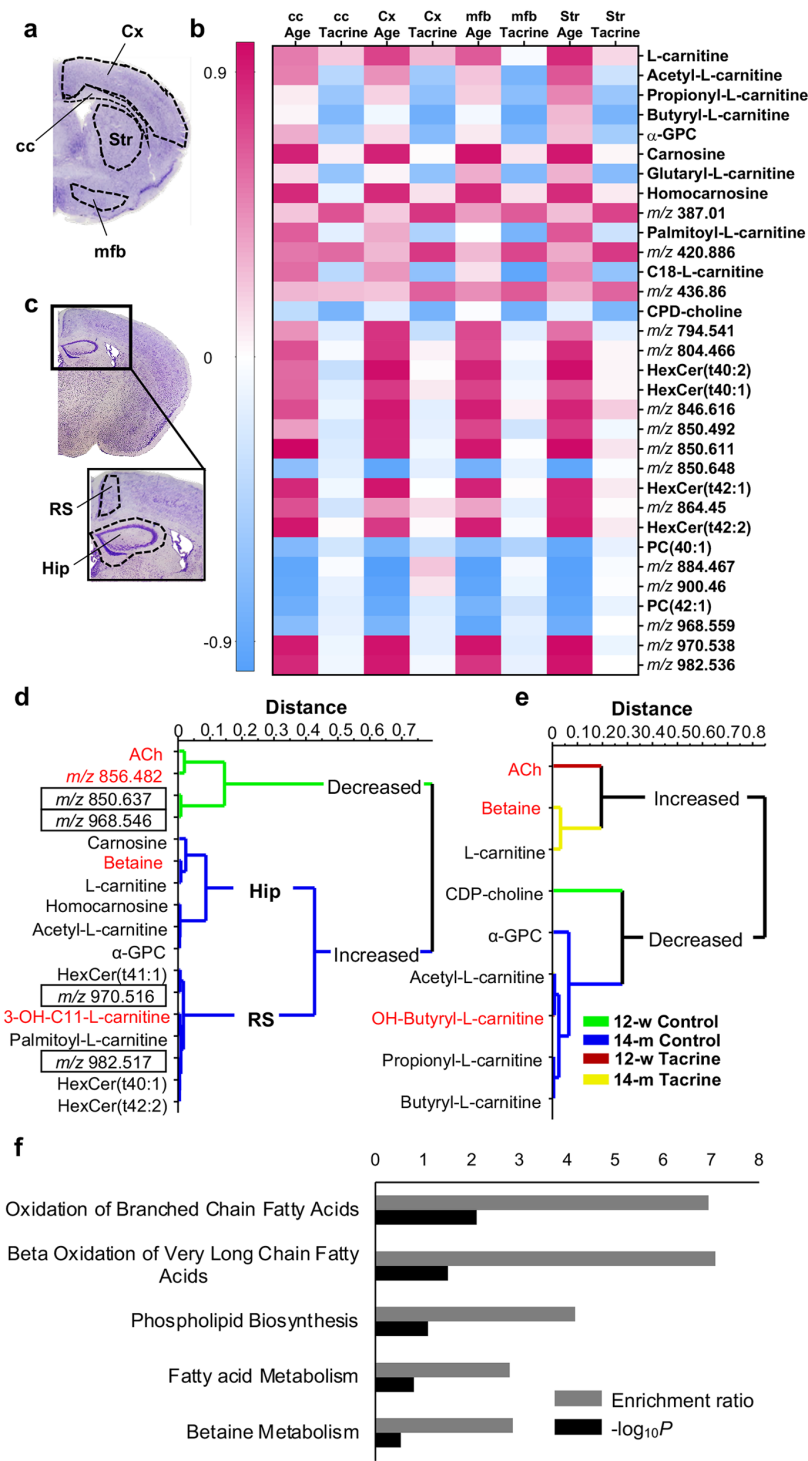
Multivariate analysis of MALDI-MSI data on the effect of age and tacrine administration in multiple brain regions. (a) The brain areas included in the first MVA are illustrated on a Nissl-stained coronal mouse brain tissue section (0.26 mm from bregma). (b) Pearson correlation coefficient (*r*) between the significantly modified analytes and the investigated age and tacrine presented as a heat map: The red palette indicates positive correlation, whereas the blue palette indicates negative correlation. (c) The brain areas in the second MVA are illustrated on a Nissl-stained coronal mouse brain tissue section (−1.06 mm from bregma). (d) Hierarchical clustering analysis of the age-induced metabolic changes. The green branches correspond to metabolites with higher log intensity values in 12-w animals, whereas the blue branches correspond to molecules with higher log intensity values in 14-m animals. Values (*m*/*z*) annotated in black boxes correspond to the following *m*/*z* values presented in the heat map: 850.648, 968.559, 970.538, and 982.536 (in order of appearance; mass difference is owing to separate tuning of the MS methods, with the second analysis being optimized for a lower *m*/*z* range). (e) Hierarchical clustering analysis of tacrine-induced metabolic changes. Green branches correspond to metabolites showing higher log intensity values in the 12-w control group, blue branches correspond to metabolites showing higher log intensity values in the 14-m control group, red branches correspond to metabolites showing higher log intensity values in the 12-w tacrine group, and yellow branches correspond to metabolites showing higher intensity in the 14-m tacrine group. Metabolites not detected in the previous PCA model are highlighted in red. (f) Enrichment analysis showing five metabolic pathways based on significance rank (i.e., altered and identified metabolites). Abbreviations: cc, corpus callosum; Cx, cortex; mfb, medial forebrain bundle; Str, striatum; α-GPC, α-glycerophosphocholine; CDP-choline, cytidine diphosphate choline; HexCer, hexosylated ceramide; PC, phosphatidylcholine; Hip, hippocampus; RS, retrosplenial cortex; ACh, acetylcholine; 3-OH-C11-l-carnitine, 3-hydroxyl-undecanoyl-l-carnitine; −log_10_ *P*, negative logarithm of the probability *P*.

Initially, MVA was performed on imaging results generated from coronal brain sections of bregma level 0.26 mm (Figure 1a and Figure S1). After model optimization (see Statistical Analysis and Figure S1), 32 metabolites were found to be significantly altered (Figure 1b). Among these, 14 were identified by tandem MS (MS/MS) analysis (Figures S2–10) and three by exact mass determination in combination with their reported lateral distribution in the brain (Table S1). Several molecules exhibited significant age- or tacrine-induced alterations, in particular carnosine and homocarnosine, hexosylated ceramides (HexCer), phosphatidylcholines (PC), l-carnitine and its acylated derivatives, as well as the choline metabolites, such as l-α-glycerophosphocholine (α-GPC) and CDP-choline (Figure 1b). The performance of the significant molecules in classifying the samples based on their age (i.e., 12-w and 14-m) was further validated with multivariate receiver operating characteristic (ROC) analysis in the two saline/tacrine administration groups separately (Figure S11).

Next, MVA was performed on coronal brain sections of bregma level −1.06 mm focusing on Hip and RS (Figure 1c). The two factors, aging and tacrine, were investigated separately. This analysis confirmed the findings of the previous modeling. Five additional metabolites were detected, of which four were identified either by MS/MS or accurate mass (Table S1). Hierarchical clustering analysis (HCA) was applied on the significantly altered metabolites, and the dendrogram of their grouping pattern (Figure 1d) was compared with the corresponding loading plot. As reflected by the dendrogram (Figure 1d), lipophilic molecules, such as longer-chain acylcarnitines and HexCers, were associated with RS.

Investigation of the tacrine effects in Hip and RS uncovered that tacrine-administered samples were significantly grouped according to age (Figure S12), which was also reflected in the different clustering of the tacrine-upregulated metabolites, that is, ACh, l-carnitine, and betaine (Figure 1e). The significantly altered metabolites were further validated by two-way ANOVA and significance refers to *P* < 0.05 (Tables S2 and S3).

The identified metabolites were used for the detection of the major metabolic pathways with enrichment analysis based on an online available library (Figure 1f).^16^ Lipid-related biochemical processes, such as fatty acid oxidation and phospholipid biosynthesis, and betaine metabolism were ranked as the top five altered pathways (Figure 1f).

### Carnosine and α-Tocopherol Are Elevated by Normal Aging in the Brain

**Figure 2:**
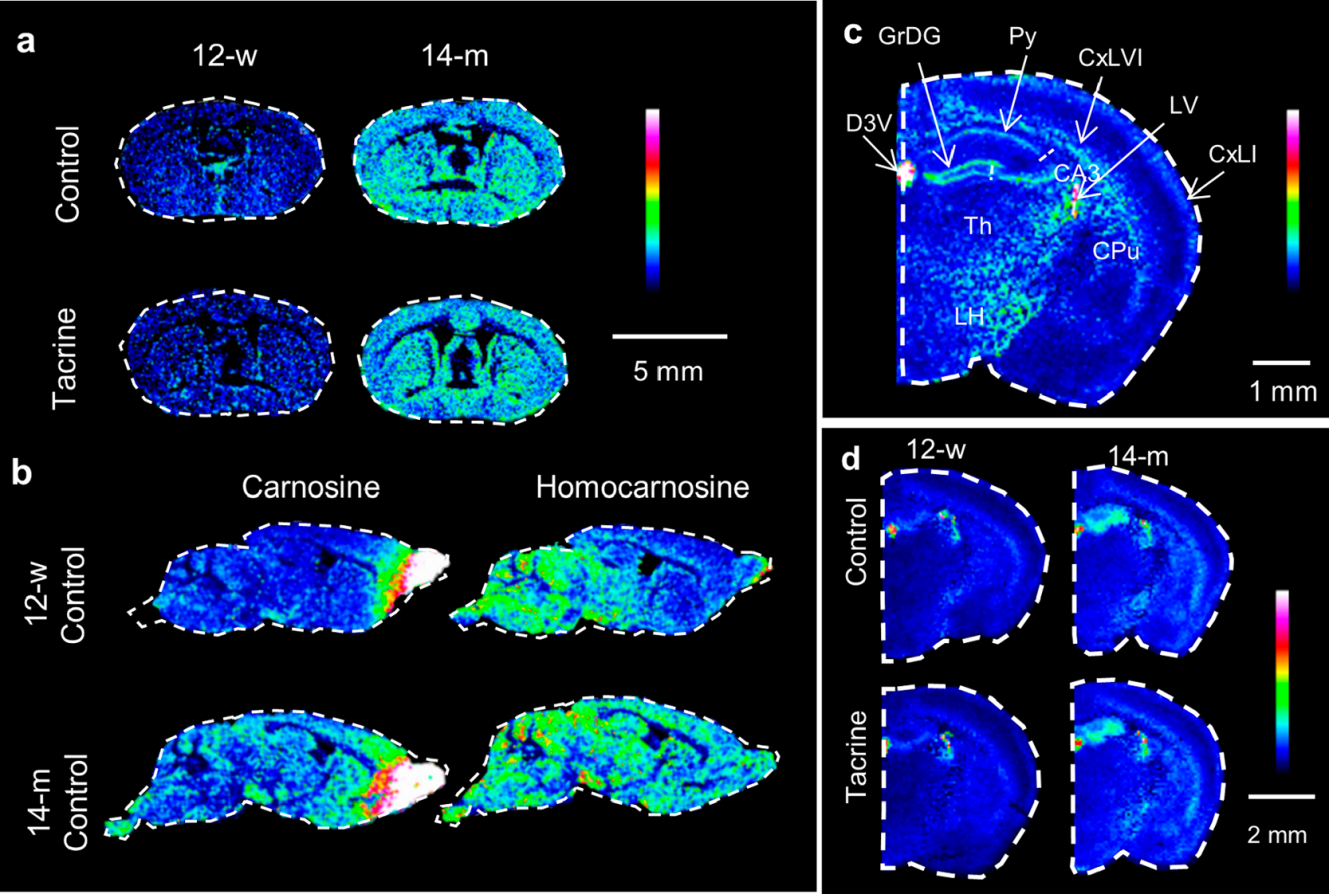
Age-induced alterations of carnosine, homocarnosine, and α-tocopherol in mouse brain tissue sections. (a) MALDI-MSI of carnosine (*m*/*z* 494.219, scaled to 100% of maximum intensity) in coronal mouse brain tissue sections (0.26 mm from bregma) at a lateral resolution of 100 μm. (b) MALDI-MSI of carnosine (scaled to 5% of maximum intensity) and homocarnosine (*m*/*z* 508.233, scaled to 60% of maximum intensity) in sagittal mouse brain tissue at a lateral resolution of 100 μm. (c) MALDI-MSI of α-tocopherol (*m*/*z* 698.492, scaled to 40% of maximum intensity) in coronal mouse brain tissue section of a 12-w control animal (−1.60 mm from bregma) at a lateral resolution of 50 μm. (d) MALDI-MSI of α-tocopherol (*m*/*z* 698.492, scaled to 100% of maximum intensity) in coronal mouse brain tissue sections (−1.06 mm from bregma) at a lateral resolution of 80 μm. The data are normalized to RMS of all data points. Abbreviations: CA3, CA hippocampal area 3; CPu, caudate-putamen; CxLI, cerebral cortex layer I; CxLVI, cerebral cortex layer VI; D3 V, dorsal third ventricle; GrDG, granular layer of the dentate gyrus; LH, lateral hypothalamus; LV, lateral ventricle; Py, pyramidal layer; Th, thalamus.

Brain levels of the dipeptide carnosine (β-alanyl-l-histidine) were significantly elevated by aging, but not affected by tacrine administration (Figure 2a, Figure S13, Table S3). In mammals, carnosine is highly accumulated in the olfactory bulb,^17^ as shown in the sagittal mouse brain sections (Figure 2b). The carnosine analog homocarnosine was also increased by aging, but did not show high accumulation in the olfactory bulb (Figure 2b,c, Table S3). Although carnosine and homocarnosine were detected in the brain by MALDI-MSI using regular matrices such as CHCA-*d*_4_ and DHB, their signal-to-noise ratio was considerably improved with the application of the FMP-10 derivatization reagent. The lack of lateral correlation between carnosine and heme b, a marker of blood vessels,^18,19^ indicates that the detected effects occur in the brain parenchyma (Figure S13). Since carnosine has been reported to possess antioxidant, antiaging, and neuroprotective properties, the age effect on a well-established free radical scavenger, that is, α-tocopherol, was investigated.^20^ In coronal mouse brain sections (at −1.60 mm from bregma, α-tocopherol showed particularly high localization in the choroid plexus of the third and lateral ventricles, the first and sixth layers of the isocortex, the caudate-putamen (CPu), the pyramidal layer of the CA3, and the granular layer of the dentate gyrus (GrDG) (Figure 2c). α-Tocopherol was significantly elevated in the hippocampal area of the older animals (Figure S13) and exhibited age-induced elevation in the first and sixth cortical layers and striatum (Figure 2d).

### l-Carnitine and Acylcarnitines: Effect of Normal Aging and Tacrine Administration

l-Carnitine was significantly elevated by aging in control animals in the Str, while the age-induced increase in the tacrine-administered group was significant in Hip, RS, and Str (Table S3). l-Carnitine was significantly elevated by tacrine in the RS, particularly in the 14-m group (Table S3). The effects of age and tacrine administration on short (acetyl-l-carnitine, propionyl-l-carnitine, butyryl-l-carnitine, hydroxybutyryl-l-carnitine, glutaryl-l-carnitine) and long fatty acid chain (OH-C11-l-carnitine, palmitoyl-l-carnitine, C18-l-carnitine) acylcarnitines were found to be compound and brain region dependent. Str and Hip showed more pronounced age effects for short chain acylcarnitines, whereas tacrine had the highest effect in mfb for both short and long chain derivatives (Figure 1 and Table S3). The age effect on the longer chain acylcarnitines, that is, palmitoyl-l-carnitine and OH-undecanoyl-l-carnitine, was considerably higher in the RS than in the Hip, especially in the saline-injected group (Figure 1d).

**Figure 3:**
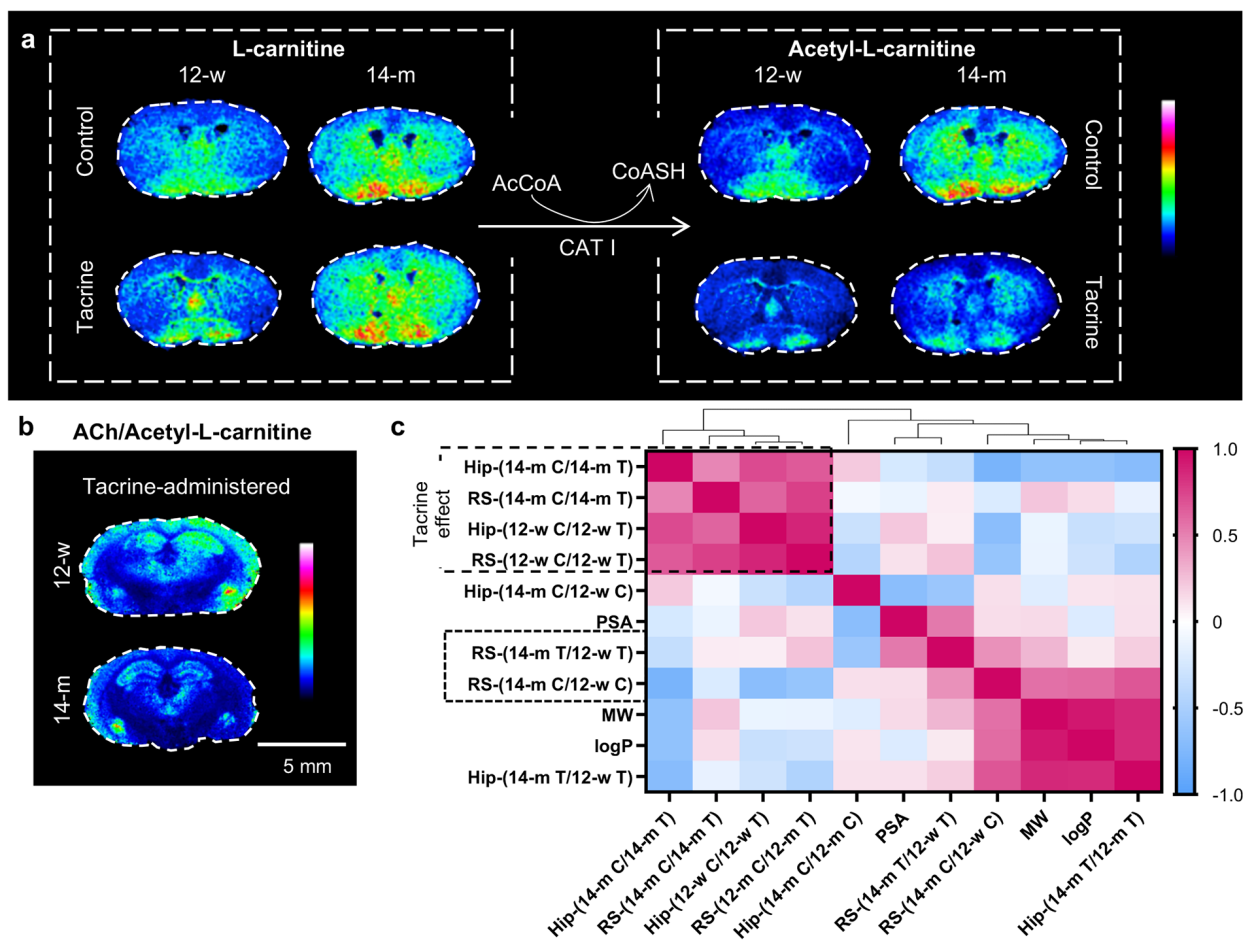
l-Carnitine/acetyl-l-carnitine pathway in coronal mouse brain tissue sections. (a) MALDI-MSI of l-carnitine (*m*/*z* 162.112) and acetyl-l-carnitine (*m*/*z* 204.123) at a lateral resolution of 100 μm (0.26 mm from bregma). The data are normalized to RMS of all data points (images scaled to 100% of maximum intensity). (b) Image of the ACh/acetyl-l-carnitine ratio at a lateral resolution of 80 μm (−1.60 mm from bregma). (c) Pearson correlation coefficient (*r*) between the molecular properties and distribution pattern of l-carnitine derivatives presented as a heat map. The red palette indicates positive correlation, whereas the blue palette indicates negative correlation. Abbreviations: AcCoA, acetyl coenzyme A; ACh, acetylcholine; C, control; CoASH, coenzyme A; CAT I, carnitine acetyltransferase I; Hip, hippocampus; log *P*, octanol/water partition coefficient (lipophilicity index); MW, molecular weight; PSA, polar surface area (hydrogen bonding index); RS, retrosplenial cortex; T, tacrine.

Acetyl-l-carnitine, the brain-abundant derivative of l-carnitine, showed significant age-induced increase in the control animals in the Str and Hip but not in the tacrine-administered group (Table S3). MALDI-MSI analysis of l-carnitine and acetyl-l-carnitine showed high accumulation in the mfb area, while the age effect was particularly pronounced in the Str, especially the dorsal part (Figure 3a). The turnover ratio of acetyl-l-carnitine to l-carnitine, as an indicator of l-carnitine metabolism, reflected the disturbance of this pathway in the Hip (compared to the 12-w control group) triggered by aging and AChE inhibition (Figure S14). The turnover ratio was significantly decreased by tacrine in 14-m mice in the area of Hip, reflecting the higher impact of the drug on this metabolic pathway in the older animals (Figure S14, Table S3). Acetyl-l-carnitine shares structural similarities to ACh and has been reported to be a potent ACh precursor by providing an acetyl moiety.^21^ In the Hip, the ion intensity ratio between ACh and acetyl-l-carnitine was significantly decreased by aging and increased by tacrine administration (Figure 3b, Figure S14).

Using untargeted *m*/*z* ratio colocalization analysis (see Imaging Analysis), different localization patterns were observed for acetyl-l-carnitine and butyryl-l-carnitine (short fatty acid chain acylcarnitine) compared to palmitoyl-l-carnitine (long fatty acid chain acylcarnitine) at brain level −1.06 mm bregma (Figure S15). Longer chain acylcarnitines, such as palmitoyl-l-carnitine, showed considerably higher localization to white matter, that is, cc and internal capsule, than to gray matter, whereas short chain analogues, for example, acetyl-l-carnitine, were more evenly distributed (Figure S15). In addition, with increasing size, that is, molecular weight, the acylcarnitines appeared to show enhanced accumulation in the RS compared to Hip in control animals of both ages (Figure S15).

The correlation between the molecular properties (i.e., molecular weight, MW; lipophilicity, log *P*; polar surface area, PSA) of l-carnitine derivatives and their regional age and tacrine effects was examined. For this, the ratios of their average intensities; 14-m control/12-w control (age-effect in the control groups), 14-m tacrine/12-w tacrine (age-effect in tacrine groups), 12-w control/12-w tacrine (tacrine effect in 12-w age groups), and 14-m control/14-m tacrine (tacrine effect in 14-m age groups) in Hip and RS were calculated. The molecular differences of the examined acylcarnitines were not strongly related to the regional tacrine effects (Figure 3c). However, in Hip the long-chain acylcarnitines (increased MW and log *P*) demonstrated stronger age-specific differences in the presence of tacrine (14-m tacrine/12-w tacrine), compared to the control group (14-m control/12-w control), while no such discrimination was observed for RS (Figure 3c).

### Age-Related Perturbation of Acetylcholine Metabolism in Response to AChE Inhibition

**Figure 4:**
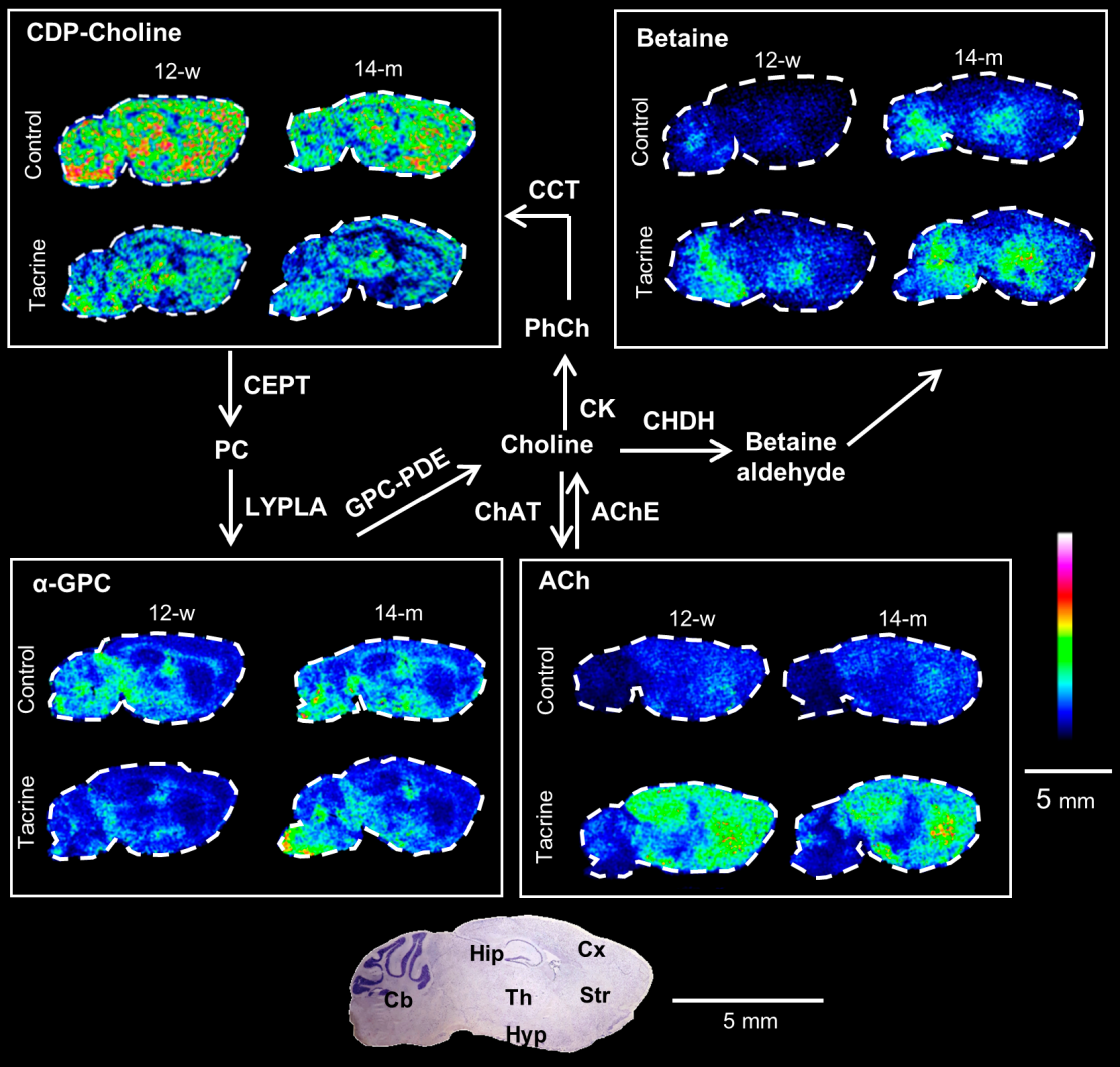
ACh/choline metabolic pathway in sagittal mouse brain tissue sections. MALDI-MS images obtained at a lateral resolution of 100 μm. The data for CDP-choline (*m*/*z* 527.069, scaled to 50% of maximum intensity), α-GPC (*m*/*z* 258.109, scaled to 100% of maximum intensity), and betaine (*m*/*z* 156.042, scaled to 100% of maximum intensity) are normalized to the RMS of all data points, whereas ACh (*m*/*z* 146.118, scaled to 60% of maximum intensity) is normalized to its internal standard.

Together with the l-carnitine pathway, the cholinergic metabolic pathway was also affected by aging and tacrine administration (Figure 4). CDP-choline and α-GPC, brain-abundant intermediate molecules of the choline pathway, were decreased by tacrine administration (Figure 4). The effect of tacrine on α-GPC was significantly higher in 14-m than 12-w animals in the area of RS (Figure S16) and mfb (Table S3), in contrast to the trend observed for ACh. In addition, in control animals, α-GPC showed an increase with age in the Hip (Figure 4b). The decreased level of CDP-choline after treatment with tacrine was significant in both age groups in all examined brain areas, such as Str (Figure S16, Table S3). Nevertheless, CDP-choline exhibited a significantly different correlation with tacrine levels depending on age in Str (Figure S16). Betaine, the final metabolite of choline metabolism, showed a high intensity in the thalamus and cerebellum (Figure 4a) and was elevated by aging, significantly after AChE inhibition in the Hip (Table S3).

### Alteration of Brain Lipids by Aging

**Figure 5:**
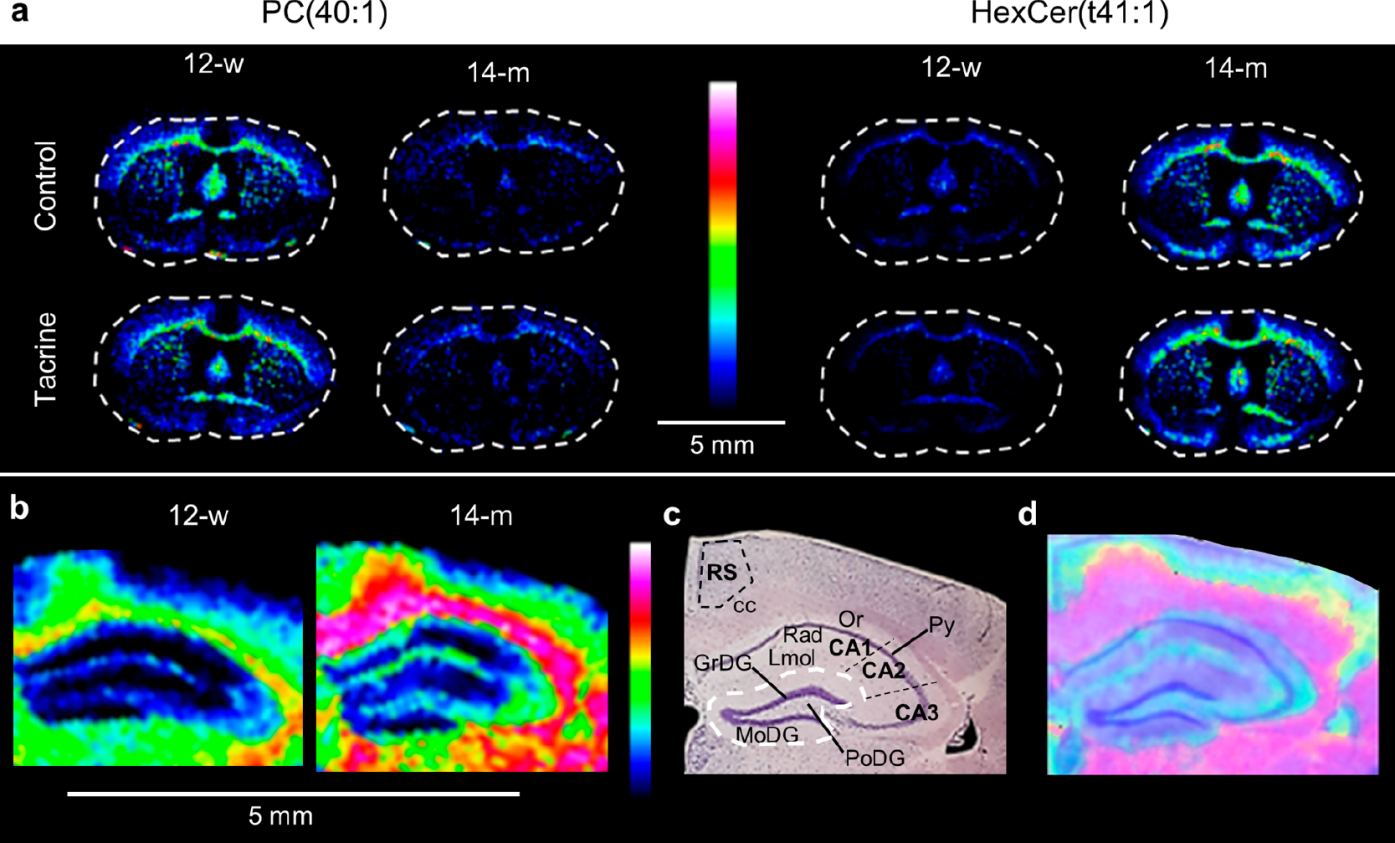
Age-modified lipid species in mouse brain tissue sections. (a) MALDI-MSI of PC(40:1) (*m*/*z* 882.631) and HexCer(t41:1) (*m*/*z* 836.654) in coronal mouse brain tissue sections (0.26 mm from bregma) at a lateral resolution of 100 μm (scaled to 100% of maximum intensity). (b) MALDI-MSI of HexCer(t41:1) (scaled to 60% of maximum intensity) in coronal mouse brain tissue sections (−1.60 mm from bregma) at a lateral resolution of 80 μm. (c) Nissl-stained mouse brain tissue section of a 14-m old animal (−1.60 mm from bregma). The DG is delineated with a white dashed line. (d) Overlay of a MALDI-MS image of HexCer(t41:1) and a Nissl-stained mouse brain tissue section.

Aging had a significant effect on brain lipids in all considered structures. PC species were decreased in older individuals, whereas HexCers (glucosylated and/or galactosylated), also known as cerebrosides, were substantially elevated (Figure 5a).

The age effect on the HexCers was higher in the RS compared to the Hip (Table S3). The age-dependent increase of the HexCers in the RS was shown to be more uniform than in Hip, where it was highly dependent on the hippocampal substructures and cellular layers (Figure 5b). HexCer (t41:1), a representative species from this class, demonstrated a higher accumulation in the oriens (Or) and lacunosum moleculare (Lmol) layers of the hippocampal proper (CA area of Hip), which are known to consist of dendrites, than in the pyramidal layer constituting the main neuronal (soma) layer of the CA^22^ (Figure 5b–d). Regarding the dentate gyrus (DG), the molecule was highly localized in the polymorphic (PoDG) layer, which contains unmyelinated axons originating from granule cells (GrDG), called mossy fibers^23^ (Figure 5b–d).

Whole brain levels of sphingosine were significantly lowered in 14-m animals, confirming age-induced metabolic disturbances of the ceramide pathway (Figure S17). Tacrine administration did not show any significant effect on the detected lipids in the investigated brain structures.

### Brain Distribution and Carrier-Mediated Transport of Tacrine and Hydroxy-tacrine

**Figure 6:**
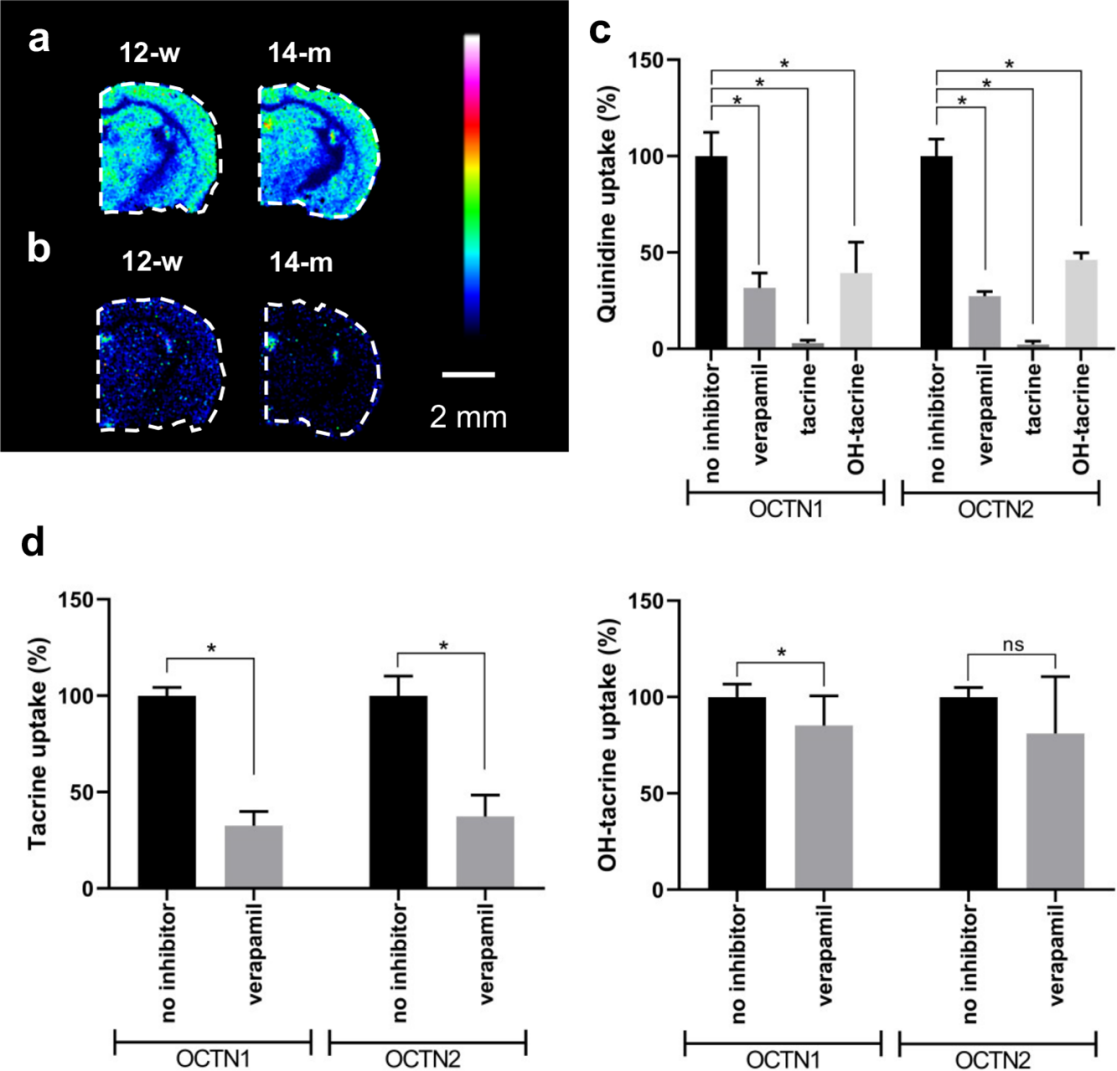
Brain distribution and OCTN-mediated uptake of tacrine and OH-tacrine. (a) MALDI-MSI of tacrine (*m*/*z* 199.122, scaled to 100% of maximum intensity) and (b) OH-tacrine (*m*/*z* 215.118, scaled to 80% of maximum intensity) in coronal mouse brain tissue sections (−1.06 mm from bregma) of 12-w and 14-m tacrine-administered animals at a lateral resolution of 80 μm. All data are normalized to 9AA, which was used as an internal standard. (c) Inhibition of OCTN1-and OCTN2-mediated uptake of quinidine by verapamil (model inhibitor), tacrine, and 1-OH-tacrine. (d) OCNT1- and OCTN2-mediated uptake of tacrine and 1-OH-tacrine. Error bars show the 95% confidence interval (*n* = 4). *P < 0.05; ns, not significant.

Tacrine was abundant in cortical areas, Hip, Str, thalamus, and hypothalamus, and its brain distribution was not significantly affected by age, consistent with our previous findings^14^ (Figure 6a). Conversely, its hydroxylated metabolites (OH-tacrine) were highly accumulated in the third and lateral brain ventricles and substantially less in the brain parenchyma, and their brain levels were significantly lower in older animals (Figure 6b).^14^ Tacrine has been reported as a possible substrate of the organic cation/carnitine transporter 2 (OCTN2) by inhibiting the OCTN2-mediated uptake of l-carnitine and acetyl-l-carnitine.^24^ Therefore, we examined whether the effects of tacrine and 1-OH-tacrine on the l-carnitine pathway could be at least partially explained by their interaction with OCTN1 and OCTN2. The *in vitro* transport experiments were performed with cell cultures overexpressing the OCTN1 or OCTN2 transporters. Inhibition experiments with the OCTN1/OCTN2 substrate quinidine showed that both investigated compounds significantly inhibited OCTN1/OCTN2-mediated quinidine uptake (Figure 6c). The effects of both compounds were compared to verapamil, a model inhibitor of OCTN transporters.^25^ The strongest inhibition was seen for tacrine (2.2%, OCTN2 and 2.9%, OCTN1 remaining uptake), followed by 1-OH-tacrine (39%, OCTN1 and 46%, OCTN2 remaining uptake). Uptake experiments further showed that tacrine is a substrate of both OCTN transporters, whereas OH-tacrine is only a substrate of the OCTN1 transporter since the uptake of both compounds was significantly reduced in the presence of the control inhibitor verapamil (Figure 6d). However, although l-carnitine and acetyl-l-carnitine significantly inhibited the uptake of quinidine (Figure S18), no significant inhibition of OCTN1 or OCTN2-mediated uptake could be observed for either l-carnitine or acetyl-l-carnitine (Figure S18).

## Discussion

In the present study, we simultaneously imaged numerous age-related neurochemical alterations in and within multiple regions of mouse brain tissue sections using high-resolution MALDI-MSI combined with machine learning models. In addition, we investigated the region-specific effects of a cognition enhancing agent, that is, the AChE inhibitor tacrine in the two age groups. The approach unveiled and visualized regional and subregional metabolic and neurochemical alterations not easily detectable using conventional tissue homogenization techniques such as LC-MS. We investigated a wide range of metabolites in multiple brain areas simultaneously with minimum sample preparation. The method also allowed visualization of the specific tissue distribution of metabolites without the need for indirect imaging approaches. Metabolic imaging investigations of the brain can partially be provided by magnetic resonance spectroscopy, a noninvasive technique used in studying normal human brain aging as well as neuropathological conditions.^26^ However, the spatial resolution and range of detectable brain metabolites are relatively narrow when using this technology, restricted mainly to the most highly abundant CNS molecules, such as *N*-acetylaspartate, choline, and creatine.^26^

Imaging of the brain levels of the dipeptide carnosine revealed novel findings in the examined brain regions, that is, carnosine levels were significantly raised by aging. Carnosine has been demonstrated to have antioxidant and age-protecting properties,^17,27^ which may indicate a potential compensatory mechanism of the brain to maintain its homeostasis. However, previous reports that analyzed carnosine in serum samples of aged rats^28^ and human blood^29^ exhibited a decline with age, highlighting the complexity of the brain influx and efflux systems. Choroid plexus and cerebral cortex have been reported to have carnosine concentrations 80- and 30-fold higher than plasma, respectively, which is indicative of active brain uptake.^30^ In addition, the brain uptake of carnosine is regulated by the peptide transporter 2 (PEPT2; SLC15A2),^30^ which is localized in the apical membrane of the choroid epithelial cells playing a key role in the efflux of small peptides and brain homeostasis.^31^ It has been reported that the clearance of a wide range of molecules from the cerebrospinal fluid, which is secreted by the choroid plexus, slows with aging^32^ as well as the paravascular clearance pathways of the brain.^33^ This could lead to an accumulation of these peptides in the CNS creating an unbalanced equilibrium between plasma and brain tissue, explaining the observed discrepancy.

An age-induced elevation of l-carnitine levels was observed in our study. l-Carnitine and acylcarnitine derivatives have been suggested as useful indicators of metabolic changes particularly connected to mitochondrial function and fatty acid β-oxidation,^34^ which was also highlighted by the pathway analysis. Str was found to be the most age-affected brain area regarding the l-carnitine pathway, for both short and long acyl chain derivatives. On the other hand, Hip and RS responded differently to age-induced alterations in this pathway depending on the acylcarnitine chain length and the presence of tacrine. In control animals, acetyl-l-carnitine was mainly elevated in the Hip, while palmitoyl-l-carnitine in the RS. These regional neurochemical differences can be associated with dissimilarities in the cell densities among the brain regions and with the distinct functions of the investigated molecules, reflecting diverse metabolic needs.^35,36^ Acetyl-l-carnitine has shown to be highly involved in transmitochondrial brain transferring of acetyl units for metabolic processes and neurotransmitter synthesis. Palmitoyl-l-carnitine has been associated with acylation of lipids and membrane interactions,^34^ processes involved in brain plasticity and neural transduction, potentially occurring in the highly myelinated RS.^37^ Unlike free l-carnitine, elevated palmitoyl-l-carnitine has been detected during apoptosis.^34^

However, in tacrine-administered animals, the age-specific differences in long-chain acylcarntines in Hip became prominent, indicating a region and age-dependent drug effect. This is an important paradigm of age-specific response to treatment in a series of metabolites belonging to the same pathway. It also reflects the complex multitarget effects of tacrine-induced AChE inhibition beyond the elevation of ACh brain levels. Imaging age-specific molecular differences between these two fine brain regions, that is, Hip and RS, manifests the powerful advantages of MSI when compared to other techniques such as LC-MS.

As both l-carnitine and acetyl-l-carnitine are reportedly antioxidant, neuroprotective, and cholinomimetic molecules,^34^ their age-related increase in the Hip and Str may be a mechanism to compensate for potential age-induced elevation of oxidative metabolic products.^38,39^ Further support to this hypothesis constitutes the recent finding of increased neuroprotective microglial phenotype and defense mechanisms with aging, despite the higher susceptibility of peripheral immune systems to neurotoxins.^40^ In addition, the significant age-induced elevation of the reportedly neuroprotective and antioxidant endogenous compounds betaine and α-tocopherol^20,41^ detected in the present study in the Hip further advocates toward this premise. We also detected high localization of α-tocopherol in the choroid plexus, a small secretory tissue found in the brain ventricles and a highly metabolically active structure with cells abundant in mitochondria, serving as an important gateway for the entrance of immune cells in the CNS.^42^

Several PCs were decreased by aging, whereas the opposite was observed for several HexCers. Although both lipid classes were highly accumulated in the fibers of the brain, such as the cc and mfb, as demonstrated by the MALDI-MSI analysis, significant age-induced alterations were detected in all examined brain areas. These findings, especially in combination with the detailed brain localization data from MALDI-MSI, are of importance since lipid signaling is reportedly involved in cellular senescence and neurodegeneration.^43^

Elevated levels of ceramides and HexCers have been discovered in senescence rodent models and AD patient samples.^43,44^ Here, we detected an age-induced increase in HexCer brain levels with simultaneous decrease in sphingosine levels. These findings indicate a considerable shift in the ceramide pathway toward glycosylation and potential loss of ceramidase (CDase) activity. The age-induced increase of HexCers was particularly large in the RS, which is a highly myelinated cortical area.^37^ In the Hip, HexCers were found to be less homogeneously distributed and accumulated in the polymorphic layer of the DG and CA3 area of the hippocampal proper, substructures playing crucial roles in learning, spatial recognition, and memory and importantly associated with aging.^45−47^

We also detected reduced levels of short- and long-chain acylcarnitine after treatment with tacrine, especially in mfb and Cx. Since there is evidence supporting the competitive inhibition of the OCTN-mediated transport of l-carnitine and acetyl-l-carnitine over the blood–brain barrier (BBB) by tacrine,^24^ we investigated the interaction of tacrine and 1-OH-tacrine with OCTN1 and OCTN2. Both molecules significantly interacted with these transporters, indicating a possible mechanism for limiting OCTN2-mediated BBB uptake of acylcarnitines, and particularly acetyl-l-carnitine.^48^ Since the major biomolecular target of tacrine is AChE, the inhibition of which leads to increased ACh brain levels, the cholinergic pathway and l-carnitine metabolism may be interrelated.^21^ Most importantly, an age-dependent difference was seen in the ACh/acetyl-l-carnitine ratio in coronal mouse brain sections from tacrine-treated animals of both the examined ages (i.e., 12-w and 14-m), especially in the hippocampal and cortical areas.

In conclusion, our results unambiguously demonstrate region-specific metabolic disturbances, such as mitochondrial dysfunction and abnormal lipid signaling, which are strongly associated with aging. The age-induced elevated levels observed for multiple endogenous antioxidants, especially in hippocampal and striatal areas, suggest that a compensatory mechanism may exist against cellular damage for the studied ages. The applied technology was able to provide a thorough insight into early stage mechanisms of normal aging, including the response to AChE inhibition, in detailed brain regions, which is important for understanding the aging process.

## Materials and Methods

### Chemicals

Deuterated analogues of acetylcholine (ACh-*d*_9_) and α-cyano-4-hydroxycinnamic acid (CHCA-*d*_4_) were obtained from CDN isotopes (Essex, UK) and Ubichem (Budapest, Hungary), respectively. Trifluoroacetic acid (TFA) and 2,5-dihydroxybenzoic acid (DHB) were purchased from Merck (Darmstadt, Germany). The solvents water, methanol, and acetonitrile were HPLC grade (VWR, Stockholm, Sweden). Cytidine diphosphate choline (CDP-choline), carnosine, acetyl-l-carnitine, 9-aminoacridine (9AA), 2,4-diphenyl-pyranylium tetrafluoroborate (DPP-TFB), and triethylamine (TEA) were obtained from Sigma-Aldrich (Stockholm, Sweden). Hydroxy-tacrine maleate was purchased from BioNordika (Stockholm, Sweden) and dl-α-tocopherol succinate was kindly provided by the lab of Prof. Per Artursson. The reactive matrix FMP-10 was produced in-house, as previously described.^49^

### Animal Experiments

Male mice (C57BL/6J) 12 weeks (12-w, *n* = 8) or 14 months (14-m, *n* = 8) old were obtained from Janvier laboratories (Scand-LAS Turku, Finland). They were housed under controlled temperature and humidity (20 °C, 53% humidity) under a 12 h light/dark cycle and fed *ad libitum*. All experiments were carried out in accordance with European Council Directive 86/609/EEC and approved by the local Animal Ethical Committee (approval no. N40/13 and N275-15). Tacrine was dissolved in saline and administered intraperitoneally (i.p.) at a dose of 10 mg/kg to both 12-w and 14-m mice. Control animals were injected with an equivalent amount of vehicle. Animals were euthanized 30 min after injection by decapitation.^50−52^ Brains were rapidly dissected out, snap-frozen in cold isopentane, and stored at −80 °C.

### Tissue Processing and Sample Preparation

Tissue sectioning was performed at −20 °C using a CM1900 UV cryostat-microtome (Leica Microsystems, Wetzlar, Germany). Coronal and sagittal brain tissue sections^15^ were cut at a thickness of 12 μm and subsequently thaw-mounted on conductive indium tin oxide-coated glass slides (Bruker Daltonics, Bremen, Germany). Coronal brain tissue sections of bregma level 0.26 mm (cortex, Cx; striatum, Str; medial forebrain bundle, mfb; corpus callosum, cc) and bregma level −1.06 mm (hippocampus, Hip; retrosplenial cortex, RS) were collected.^15^ For evaluation and confirmation of the results, coronal brain sections at bregma level −1.60 mm and sagittal brain sections were utilized. The prepared slides were stored at −80 °C. Sections were desiccated at room temperature for 15 min, then imaged optically using a photo scanner (Epson Perfection V500).

CHCA-*d*_4_ (5 mg/mL dissolved in 50% acetonitrile and 0.2% TFA) was applied with an automatic TM-sprayer (HTX-Technologies LLC, Chapel Hill, NC, USA) at 90 °C using six passes with a solvent flow rate of 70 μL/min, spray head velocity of 1100 mm/min, and track spacing of 2.0 mm. DHB (35 mg/mL in 50% acetonitrile and 0.2% TFA) was sprayed using eight passes with a solvent flow rate of 70 μL/min, a spray head velocity of 1100 mm/min, and track spacing of 3.0 mm. For both applications, a nitrogen pressure of 6 psi was used as nebulizing gas. CHCA-*d*_4_ was chosen over regular CHCA matrix to avoid overlapping matrix signals when applied on tissue.^53^

For MALDI-MSI of ACh, prior to application of the matrix CHCA-*d*_4_, a solution of ACh-*d*_9_ (0.367 μM) in 50% acetonitrile and 0.2% TFA was applied with the same TM-sprayer method as the matrix application.

DPP-TFB and FMP-10 derivatization was performed as described previously.^49,54^ Briefly, DPP-TFB was dissolved in 7.2 mL of 75% methanol and alkalified with 3.5 μL of TEA to obtain a 1.3 mg/mL derivatization solution. FMP-10 was dissolved in 70% acetonitrile to a concentration of 1.8 mg/mL. Both matrices were sprayed on the mouse brain tissue sections using the TM-sprayer at 80 °C with a flow rate of 80 μL/min for 30 passes, velocity of 1100 mm/min, 2 mm track spacing, and 6 psi nitrogen pressure.

### MALDI-MSI Analysis

All MALDI-MSI experiments were performed in positive ionization mode using a MALDI-FTICR (Solarix XR 7T-2ω, Bruker Daltonics) mass spectrometer equipped with a Smartbeam II 2 kHz laser. The size of laser was chosen to give a lateral resolution of 80–100 μm, and the instrument was tuned for optimal detection of small molecules (*m*/*z* 150–1000 and *m*/*z* 86–1000 for inclusion of ACh) using the quadrature phase detection (QPD) (2ω) mode. When the CHCA-*d*_4_ matrix was used, the time-of-flight (TOF) value was set at 0.600 ms and the transfer optics frequency at 6 MHz. The quadrupole isolation *m*/*z* value (Q1 mass) was set at *m*/*z* 200.00. As lock masses, the CHCA-*d*_4_ matrix ions ([M + H]^+^ 194.074977, [2M + H]^+^ 387.142677, [2M + K]^+^ 425.098558), and phosphatidylcholine 34:1 ([M + H]^+^ 760.58508) were used. Spectra were collected by summing 80 laser shots per pixel. For analysis of DHB prepared samples, the Q1 mass was *m*/*z* 250 and the matrix ions ([M + H]^+^*m*/*z* 153.033885, [2M – 2H_2_O + H]^+^*m*/*z* 273.039364, [3M – 3H_2_O + H]^+^*m*/*z* 409.055408), and phosphatidylcholine 34:1 ions ([M + H]^+^*m*/*z* 760.58508) were used as lock masses. The TOF and transfer optics frequency values remained the same. Spectra were collected by summing 100 laser shots per pixel. Both methods were calibrated with red phosphorus over an appropriate mass range. The laser power was optimized at the start of each analysis and then held constant during the MALDI-MSI experiment. Any possible bias due to factors such as matrix degradation or variation in mass spectrometer response was minimized by randomized analysis of the tissue sections.

For targeted MALDI-MSI experiments on specific molecular classes, such as ACh, l-carnitine and acetyl-l-carnitine, tacrine, and high molecular weight lipids, continuous accumulation of selected ion (CASI) was used to improve the limit of detection of these analytes. For ACh, l-carnitine, and acetyl-l-carnitine, the Q1 mass was set at *m*/*z* 180 with a mass window of 80 Da. The TOF and frequency values were adjusted to 0.550 ms and 4 MHz, respectively. For tacrine and OH-tacrine, the Q1 mass was set at *m*/*z* 199 with a mass window of 50 Da, the TOF was 0.550 ms, and the frequency was 6 MHz, as previously described.^14^ For lipids, the Q1 mass was set at *m*/*z* 838.60 with a mass window of 40 Da, and the TOF and frequency values were adjusted to 0.750 ms and 4 MHz, respectively. For MALDI-MSI of FMP-10 derivatized carnosine and α-tocopherol and DPP-TFB derivatized sphingosine (d18:1), methods were used as previously described.^49,54^

For tissue and standards, when available, MALDI-tandem MS (MS/MS) experiments were performed by isolating the precursor ion in a mass window of 1 or 2 Da and allowing the target ions to be selected in the quadrupole and fragmented in the collision cell. The collision energy voltage was optimized for every analyte and varied between 10.0 and 35.0 V. Following MALDI-MSI analysis, the sections were histologically analyzed using Nissl staining.

### Imaging Analysis

MSI data were visualized in FlexImaging (v. 5.0, Bruker Daltonics). For further analysis, data were imported to SCiLS Lab (v. 2019a Pro, Bruker Daltonics), and brain regions were annotated according to a stereotaxic atlas.^15^ Four experimental groups were considered for the analysis: 12-w control, 14-m control, 12-w tacrine-treated, and 14-m tacrine-treated. A MALDI-MSI experiment performed by applying CHCA-*d*_4_ on coronal brain sections (*n* = 4) of bregma level 0.26 mm was used for the initial untargeted analysis. Four discrete brain areas were investigated: cc, Cx, mfb, and Str. All individual spectra were normalized to the root-mean-square (RMS) of all data points. The ion at *m*/*z* 146.1176, corresponding to ACh, was normalized to the internal standard ACh-*d*_9._ The maximum ion intensities of the 2500 most intense peaks of the average spectra from each brain region in the mass range *m*/*z* 150–1000 were exported from SCiLS for statistical analysis. The average intensity values per brain area were log transformed. Using the same process, data from Hip and RS of coronal sections of brain level −1.06 mm (*n* = 4) were also analyzed by applying CHCA-*d*_4_. In these cases, the mass range was *m*/*z* 86–1000 to include small molecules, such as ACh which was normalized to the internal standard (ACh-*d*_9_). Experiments performed by applying DHB on coronal (*n* = 4, bregma level 0.26 mm), and sagittal (*n* = 3) brain sections were used for validation and complementary analysis. These data were acquired as described above. For MALDI-MSI of tacrine and its hydroxylated metabolite(s), 9AA was used as an internal standard for normalization.^14^

Untargeted spatial *m*/*z* colocalization analysis, implemented in SCiLS Lab, was used to elucidate *m*/*z* values exclusively colocalized with a given *m*/*z* image. The method used Pearson’s correlation and considered only statistically significant interactions (*P* < 0.05). This approach was applied for acetyl-l-carnitine and palmitoyl-l-carnitine. The lateral distribution of heme b (*m*/*z* 616.177) was used for the estimation of brain vasculature.^18,19^

### Machine Learning Models

The simultaneous investigation of two two-level factors (i.e., two different age points and saline/tacrine administration) in multiple brain regions with a multidimensional data generating technique as MSI made the application of conventional statistical methods of hypothesis testing challenging. Therefore, unsupervised and supervised multiclass classification, correlation, and regression algorithms were applied for data visualization and better exploration.

MVA was performed using SIMCA v.13.0 (Sartorius Stedim Biotech, Umeå, Sweden). Because all included variables were of the same type, that is, log-transformed ion intensities, centering and autoscaling to unit variance (the SIMCA default scaling option) were considered adequate. PCA was initially applied to obtain an overview of the data and enable identification of possible outliers. The Hotelling T^2^ ellipse (T2Crit) and distance to model (DModX) at 95% confidence interval were used as criteria for outlier detection. Subsequently, PLS-DA was implemented to reveal specific alterations among the four groups. In PLS-DA, a dummy *Y* variable was assigned to every defined class, corresponding to the response variable. The number of components and number of original variables *X* included in the model were defined after evaluation of the fit according to *R*^2^, *Q*^2^, and classification performance, that is, extent of group separation as presented in the score plot. In general, *Q*^2^ values >0.5 were considered necessary for the model establishment, while a difference lower than 0.2 between the *R*^2^ and *Q*^2^ was taken into account for obtaining reliable models. The variable selection process was based on the variable influence on projection (VIP) and loading value of every variable in each component. Terms with VIP> 1 were considered most relevant for explaining *Y*, which, in the case of PLS-DA, corresponded to the different classes. A “bottom-up” HCA was performed on the final PLS-DA model for grouping the original variables (metabolites) using the Ward algorithm to calculate distances.

The final model was validated based on the misclassification table (i.e., number of false positives and false negatives given the number of correct predictions at a probability level of 0.35) and the permutation test (100 permutations) (Table S4). Finally, PLS-DA models were converted into PCA as another approach of validation.

Multivariate ROC analysis was applied to validate the age classification performance of the significant molecules at 0.26 mm from bregma. ROC curves were generated by Monte Carlo cross validation using balanced subsampling. In each validation, 2/3 of the samples were used to evaluate feature importance, and the remaining 1/3 were used to validate the models created with the first step. The multivariate algorithm used for ROC curve analysis was support vector machines.

The correlations between the significant metabolites and the investigated factors (i.e., age, tacrine) were obtained with Pearson *r* correlation coefficient and usually visualized as heatmaps. Linear regression was applied to explore the linear relationship (*R*^2^) between two specific variables.

### Univariate Statistical Analysis

One- and two-way ANOVA with a Tukey’s post hoc test and significance level (α) set at 0.05 were used to verify the MVA results (SPSS v. 25.0, IBM, Armonk, NY, USA) (Table S3). Together with the *F* statistics and the *P* value, the effect size expressed as partial η^2^, i.e., the proportion of total variability attributable to a factor such as the age and the tacrine administration, and the observed power, that is, the power of the test when the alternative hypothesis is set based on the observed value, were also considered. Normality and homogeneity of the data were examined with the Shapiro-Wilk and Levene’s test, respectively. GraphPad Prism 5 software was used for the illustration of dot plots based on one-way ANOVA using Tukey’s multiple comparison test (*P* < 0.05).

### Identification of Metabolites

The ions that displayed significant alterations by aging and/or tacrine were primarily identified by database searches (www.hmdb.ca,^55^www.lipidmaps.org,^56^ and metaspace2020.eu(57)) based on the high mass accuracy provided by the FTICR MS analysis. Subsequently, standards were used to confirm the identifications. MALDI-MS/MS was performed on tissues, and the product ions were compared to product ion spectra of standards or previously published data. In the case of MS/MS imaging, their brain tissue distribution of the product ions was compared to the distribution of the precursor ion (Table S1, Figures S2–S10). In cases where ions present in brain tissue were of very low abundance, identification was based on mass accuracy and high correlation and colocalization (*r* > 0.5, *P* < 0.05) with already identified metabolites. Selectivity of reactive matrices, that is, DPP-TFB and FMP-10, toward primary amines and/or phenolic hydroxyls was used to identify species bearing such functional groups.

### Metabolite Set Enrichment Analysis

The detection of major pathways associated with the identified metabolites was performed using the online MetaboAnalyst platform.^16^ The applied library was the pathway-associated metabolite sets consisting of 99 metabolites. The enrichment ratio was defined as the ratio of the observed count of hits, that is, detected metabolites per pathway to the count expected by chance.^16^

### *In Vitro* Transport Experiments

For the cell culture, human embryonic kidney 293 (HEK293) Flp-In cells stably overexpressing the OCTN1 or OCTN2 transporters (kindly provided by Professor Kathleen Giacomini, University of California, San Francisco)^58,59^ were cultured in Dulbeccos’s modified Eagle’s medium supplemented with 10% fetal bovine serum, 1% penicillin and streptomycin solutions (100 units/mL penicillin and 100 mg/mL streptomycin), and 2 mM l-glutamate supplemented with 75 μg/mL of hygromycin B. The cell culture media and supplements were from ThermoFischer Scientific (Waltham, MA, USA) or Sigma-Aldrich (St. Louis, MO). Cells were cultured under 37 °C, 95% humidity and 5% CO_2_ and subcultured twice a week.

Four days prior to the experiments, cells were seeded in 24-well CellBind plates (Corning, Amsterdam, Netherlands) at a density of 600,000 cells/well. Twenty-four h before experiments, the cell culture medium was removed and replaced with fresh culture medium. On the day of the experiments, cells were washed twice with prewarmed Hank’s balanced salt solution (pH 7.4), followed by incubation at 37 °C with prewarmed test solutions. The experiments were terminated by adding cold phosphate buffered saline, followed by two washing steps. For inhibition experiments, cells were incubated with a solution containing 1 μM quinidine with or without 500 μM l-carnitine, acetyl-l-carnitine, tacrine, or OH-tacrine for 10 min. Verapamil, also at 500 μM, was used as an inhibitor control. For uptake experiments, cells were incubated with a solution containing 1 μM l-carnitine, acetyl-l-carnitine, tacrine, or OH-tacrine with or without 500 μM verapamil for 10 min. All experiments were performed in at least triplicate on two independent occasions. One-way ANOVA with Dunnett’s multiple comparison test implemented in GraphPad Prism 8.1.0 (GraphPad Software, San Diego, CA) was used for statistical comparisons.

After the final washing steps, the cells were dried and extracted using 0.2 mL acetonitrile:water 60:40 spiked with 50 nM warfarin as an internal standard. For analysis of quinidine, tacrine, and OH-tacrine, this was followed by centrifugation at 3000×*g* for 20 min at 4 °C using a 5810R centrifuge from Eppendorf (Hamburg, Germany). For analysis of l-carnitine and acetyl-l-carnitine, the extracted samples were evaporated using a GeneVac EZ-2 plus (Genevac Ltd., Ipswich, Suffolk, UK) at 40 °C until dryness, reconstituted in 0.2 mL Milli-Q H_2_O, followed by centrifugation at 3000×*g* for 20 min at 4 °C. After centrifugation, all supernatants were injected for UPLC-MS/MS analysis (Acquity UPLC-TQ MS, Waters Corp., Milford, MA) of intracellular accumulation of the investigated compounds. Compounds were analyzed in positive electrospray mode operating at 2 kV with 500 °C desolvation temperature in the ion source. l-Carnitine transitions in the MRM mode were 161.92 > 59.93 and 161.92 > 102.82 (cone 22 V, collision energy (CE) 16 eV), acetyl-l-carnitine transitions were 203.92 > 59.82 (cone 22 V, CE 16 eV) and 203.92 > 84.79 (cone 22 V, CE 22 eV), tacrine transitions were 198.92 > 143.87 (cone 50 V, CE 34 eV) and 198.92 > 170.85 (cone 50 V, CE 28 eV), OH-tacrine transitions were 214.91 > 180.86 (cone 22 V, CE 34 eV) and 214.91 > 197.00 (cone 22 V, CE 30 eV), and quinidine transitions were 325.16 > 78.50 (cone 36 V, CE 32 eV) and 325.16 > 81.42 (cone 36 V, CE 38 eV). First transitions for each compound were used for quantification, whereas second transitions were used as a qualifier ion. The transition in MRM mode for the internal standard warfarin was 309.00 > 163.00 (cone 22 V, CE 14 eV). The compounds were separated on a BEH C18 2.1 × 50 mm, 17 μm column (Waters Corp., Milford, MA) and eluted with mobile phase A1 (5% acetonitrile and 0.1% formic acid in H_2_O) and B1 (0.1% formic acid in acetonitrile) with a linear gradient starting from 5% B1 at 0.5 min and increasing to 90% B1 at 1.2 min with (quinidine) or with mobile phase A2 (0.05% heptafluorobutyric acid and 0.05% propionic acid in H_2_O) and B2 (0.05% heptafluorobutyric acid and 0.05% propionic acid in acetonitrile) with a linear gradient starting from 5% B2 at 0.5 min and increasing to 100% B2 (l-carnitine, acetyl-l-carnitine, tacrine, OH-tacrine) at 1.2 min. The total analysis time was 2 min, and the injection volume was 5 μL per sample. Total protein content was measured using the BCA Protein Assay Reagent Kit (Pierce Biotechnology, Rockford, IL) according to the manufacturer’s instructions, and the resulting protein concentrations were used for normalization of the cellular uptake.

## Supporting Information Available

Multivariate analysis of MALDI-MSI data, structure validation of detected metabolites by MS/MS analysis, statistical approaches and MSI data corroborating the results (figures). List of identified metabolites, statistical data of metabolites per brain region (tables) (PDF)
