# Supplementary material for: Integration of Mass Spectrometry Imaging and Machine Learning Visualizes Region-Specific Age-Induced and Drug-Target Metabolic Perturbations in the Brain
Source: ACS Chem Neurosci. 2021 May 3;12(10):1811–23. doi: 10.1021/acschemneuro.1c00103 (PMC8291481; doi:10.1021/acschemneuro.1c00103)
Supplement: Supplementary file 1 — cn1c00103_si_001.pdf [file cn1c00103_si_001.pdf]

Supporting Information

Integration of Mass Spectrometry Imaging and Machine Learning Visualizes Region-Specific Age-Induced and Drug-Target Metabolic Perturbations in the Brain

Theodosia Vallianatou[1], Reza Shariatgorji[1,2], Anna Nilsson[1,2], Maria Karlgren[3], Heather Hulme[1], Elva Fridjonsdottir[1], Per Svenningsson[4], Per E. Andrén[1,2]*

1. Department of Pharmaceutical Biosciences, Medical Mass Spectrometry Imaging, Biomedical Centre 591, Uppsala University, SE-75124 Uppsala, Sweden
2. Science for Life Laboratory, Spatial Mass Spectrometry, Biomedical Centre 591, Uppsala University, SE-75124 Uppsala, Sweden
3. Department of Pharmacy, Uppsala Drug Optimization and Pharmaceutical Profiling (UDOPP), Biomedical Centre 580, Uppsala University , SE-75123 Uppsala, Sweden
4. Department of Clinical Neuroscience, Section of Neurology, Karolinska Institutet, SE-17177 Stockholm, Sweden

* Corresponding author, email: per.andren@farmbio.uu.se

Content

Figures S1–18 p. S2 – S18
Tables S1–4 p. S19 – S30
References p. S31

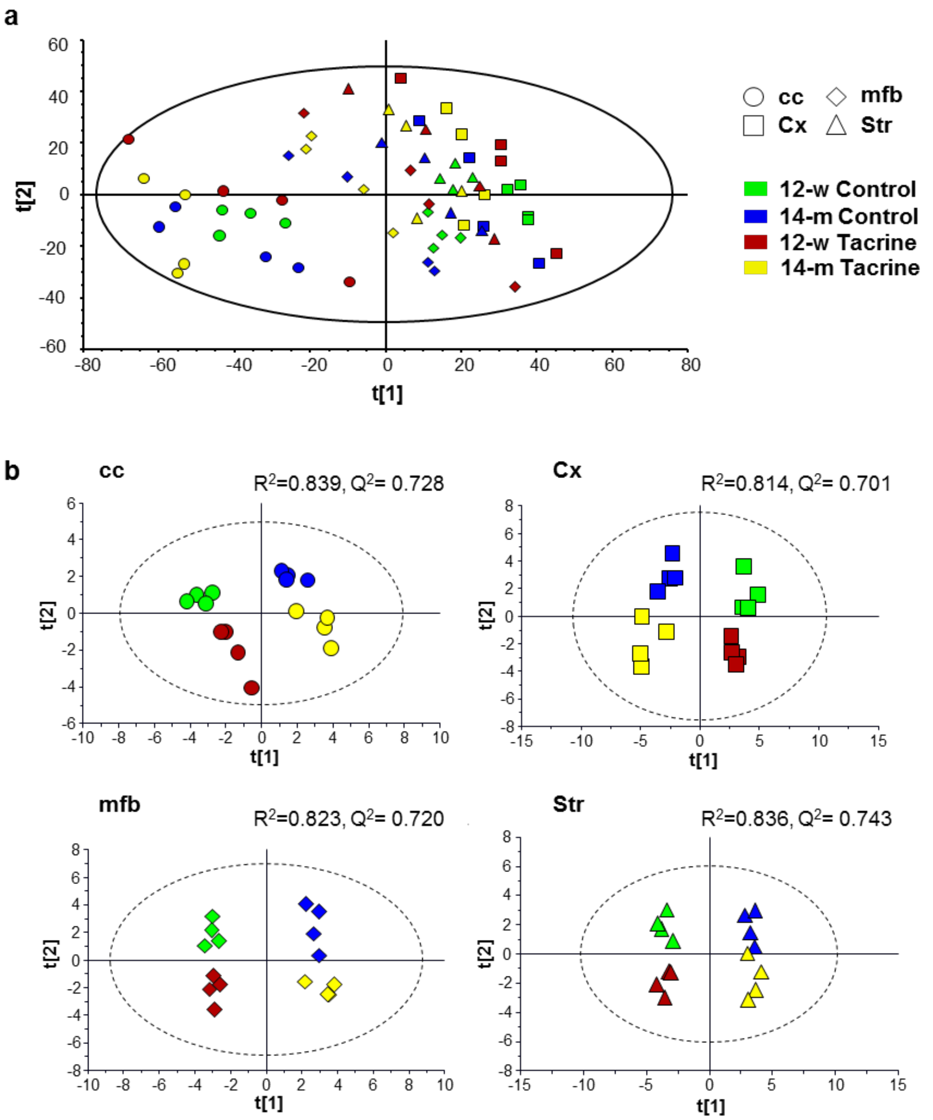

Figure S1. Multivariate analysis of MALDI-MSI data of brain tissue sections at 0.26 mm from bregma.

a) Principal component analysis (PCA) including the studied brain structures and 2,500 of the most intense m/z values showed a high influence of the different brain areas and lack of strong outliers, with the 12-w control group exhibiting the strongest clustering tendency. **b**) Different partial least squares-discriminant analysis (PLS-DA) models were developed for each brain area. The models obtained after optimization, resulting in an average of 20 ± 5 m/z values per model, were converted into PCA as an indicator of model reliability [1] and subsequently used for interpretation. For each examined brain region, t-scores of the first principal component (t[1]) showed significant discrimination between the two investigated ages (two-way ANOVA with Tukey's post hoc test, P<<0.001), as illustrated in the score plots. This indicated a strong aging effect on the included small brain molecules and metabolites as the first component explained the main variance in the data (50-62%). On the other hand, the second component (t[2]) significantly separated the samples according to the treatment (two-way ANOVA with Tukey's post hoc test, P<<0.001).

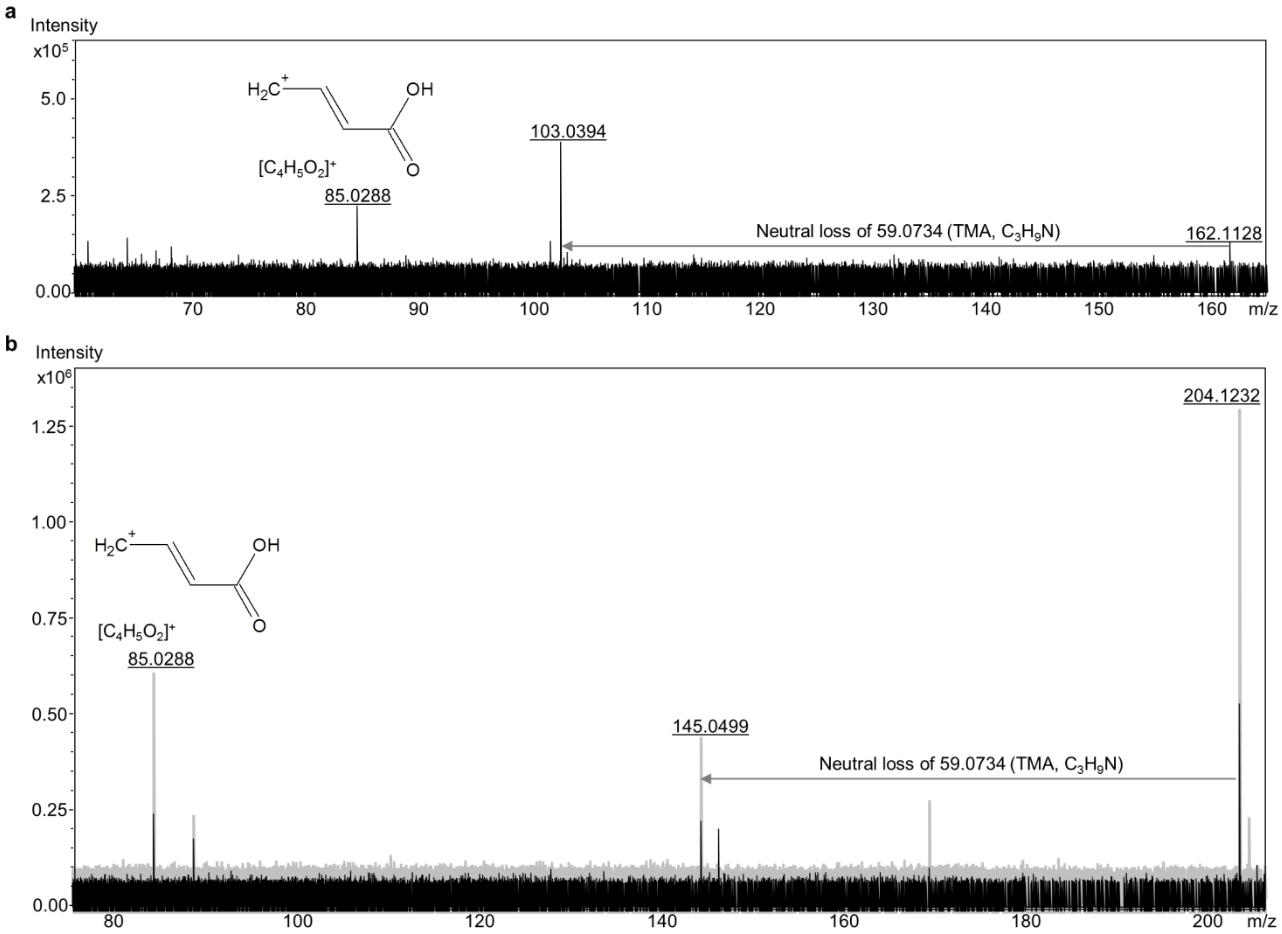

Figure S2. MALDI-MS/MS spectra of L-carnitine (*m/z* 162.1128, precursor ion) and acetyl-L-carnitine (*m/z* 204.1232, precursor ion) from the corpus callosum of a mouse brain tissue section (black) and standard solution of acetyl-L-carnitine (grey).

a) L-carnitine, the precursor ion (*m/z* 162.11) was isolated with a mass window of 2 Da and collision energy voltage of 20.0-25.0 V. The product ion peaks of *m/z* 103.0394 and *m/z* 85.0288 correspond to neutral loss of 59.07 Da (trimethylamine, TMA) and the key fragment $[C_4H_5O_2]^+$, respectively. The $[C_4H_5O_2]^+$ product ion is characteristic for carnitine-related molecules, as well as the neutral loss of a TMA molecule [2]. **b)** For the identification of acetyl-L-carnitine, the precursor ion (*m/z* 204.12) was isolated with a window of 1 Da and the analysis was performed on both a tissue section and acetyl-L-carnitine standard for comparison (collision energy voltage 20.0 V).

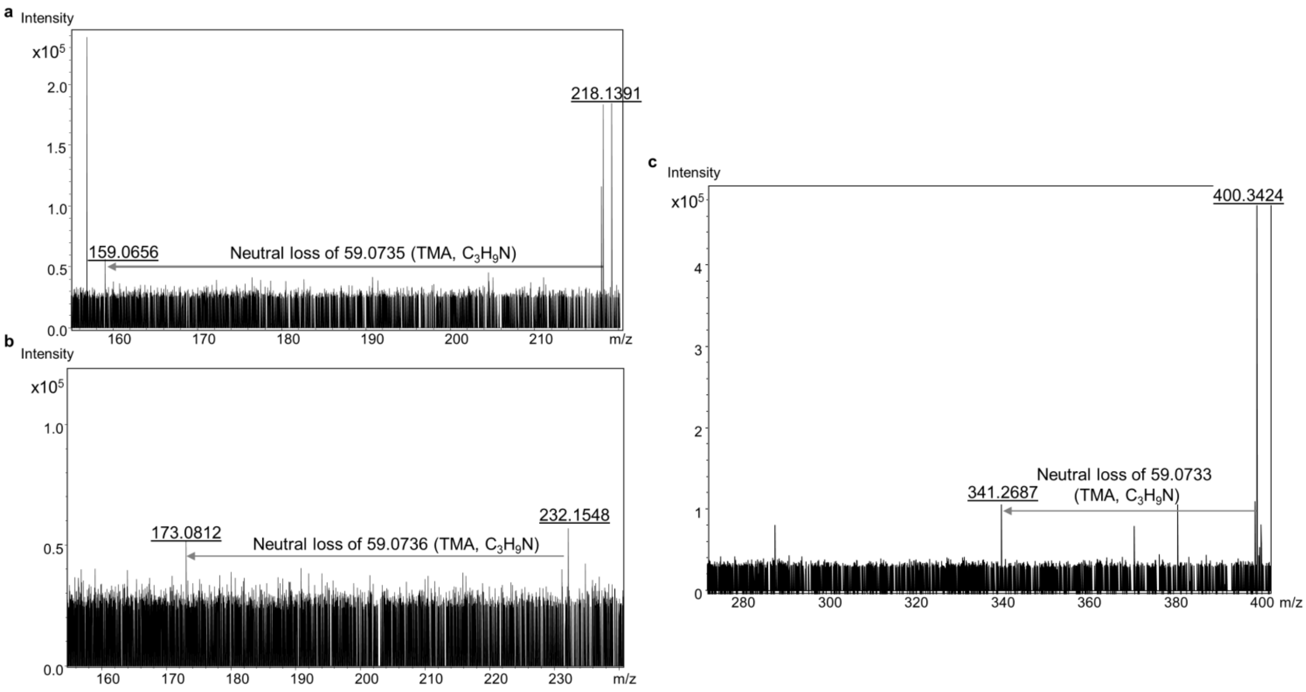

Figure S3. MALDI-MS/MS spectra of propionyl-L-carnitine (*m/z* 218.1391, precursor ion), butyryl-L-carnitine (*m/z* 232.1548, precursor ion) and palmitoyl-L-carnitine (*m/z* 400.3424, precursor ion) from the corpus callosum of a mouse brain tissue section.

For the identification of **a)** propionyl-L-carnitine, **b)** butyryl-L-carnitine and **c)** palmitoyl-L-carnitine, the precursor ions were set at *m/z* 218.50, *m/z* 232.60 and *m/z* 400.30, respectively, with an isolation window of 1 Da. The collision energy voltage values were 5V, 10V and 12V, respectively. The low abundance of the precursor molecules hampered formation of the $[C_4H_5O_2]^+$ product ion, hence the corresponding mass range is not shown. Abbreviations: TMA, trimethylamine.

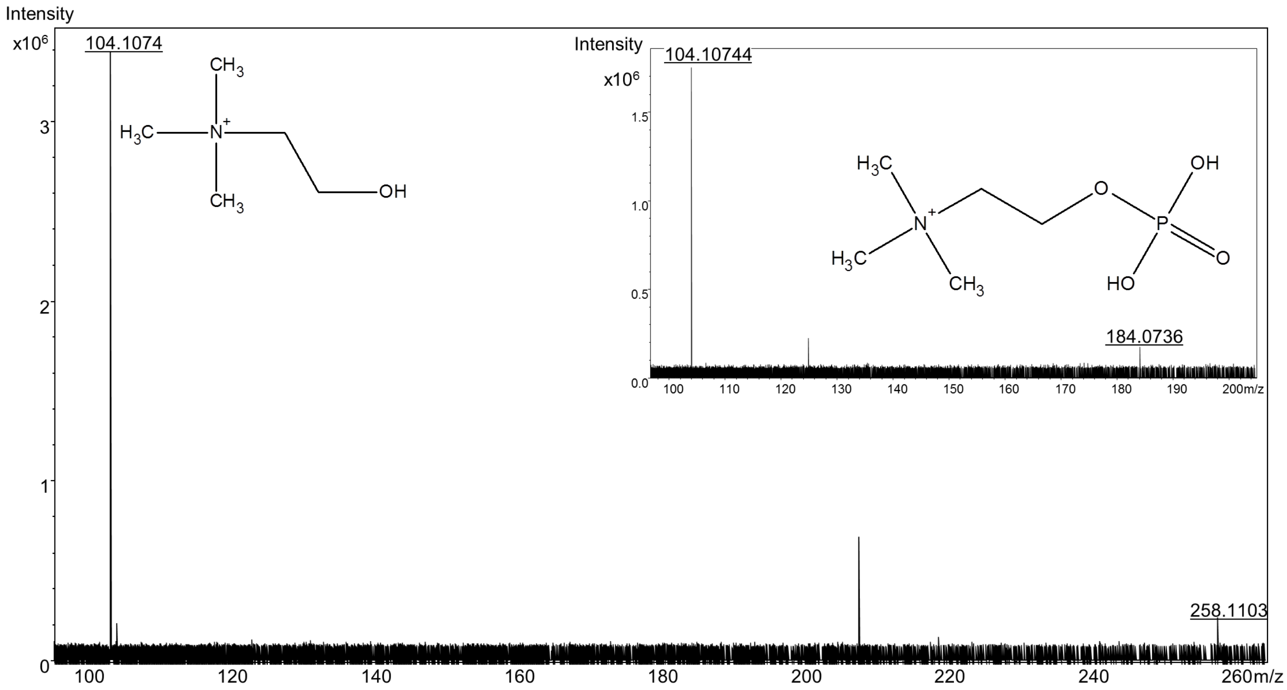

Figure S4. MALDI-MS/MS spectrum of α-GPC (*m/z* 258.1103, precursor ion) in the corpus callosum of a mouse brain tissue section.

For α-GPC, the precursor ion at *m/z* 258.11 was isolated with a mass window of 2 Da. The product ion *m/z* 104.1074, generated with collision energy voltage of 20.0-25.0 V, confirms the presence of a choline group in the precursor molecule, while the use of higher collision energy voltage energy (30.0 V) led to formation of the phosphocholine product ion (*m/z* 184.0736), confirming the identity of the precursor ion.

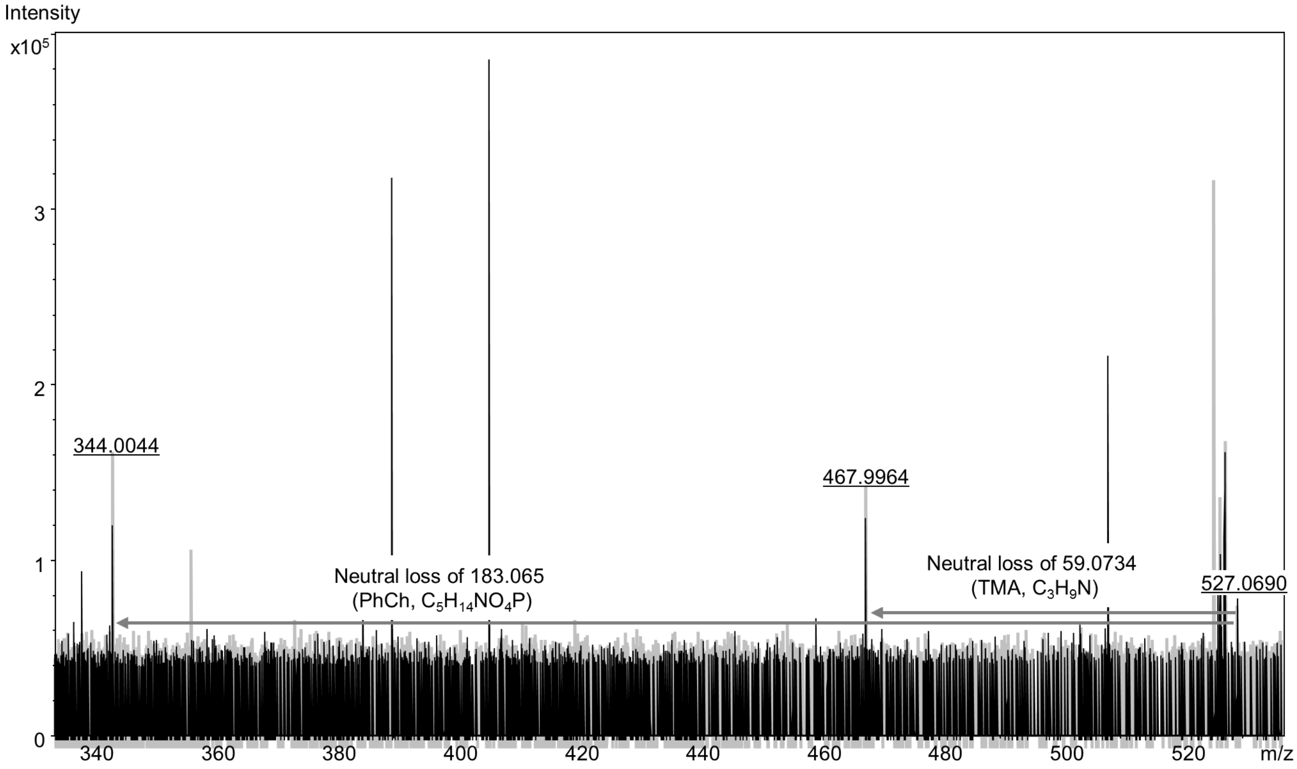

Figure S5. MALDI-MS/MS spectrum of CDP-choline (*m/z* 527.0690 ([M+K]$^+$), precursor ion) in the cortex of a mouse brain tissue section (black) and a standard solution (grey).

The precursor ion at *m/z* 527.0690 was isolated in a window of 1 Da, both in tissue and from standard solution with collision energy voltage of 20.0 V. Abbreviations: TMA, trimethylamine; PhCh, phosphocholine.

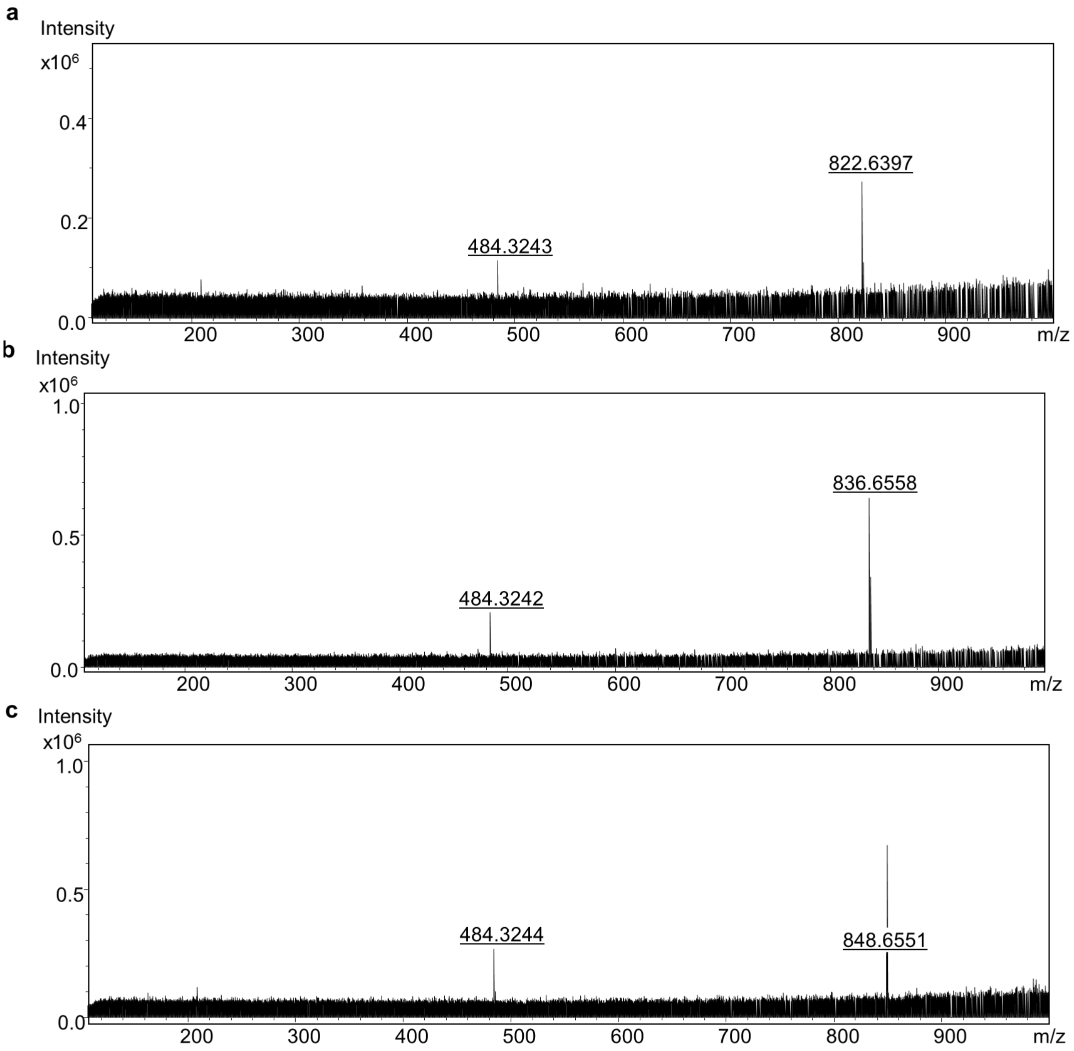

Figure S6. MALDI-MS/MS spectra of HexCer (t40:1) (*m/z* 822.6397 ($[M+Na]^+$), precursor ion), HexCer (t41:1) (*m/z* 836.6558 ($[M+Na]^+$), precursor ion) and HexCer (t42:2) (*m/z* 848.6551 ($[M+Na]^+$), precursor ion) in the corpus callosum of mouse brain tissue.

Identification of HexCers in a tissue section using MALDI-MS/MS to isolate the precursor ions, **a)** *m/z* 822.64, **b)** *m/z* 836.65 and **c)** *m/z* 848. 65 in a window of 1 Da with collision energy voltage of 20.0- 30.0 V. The product ion *m/z* 484.3243 (average value) is in good agreement with the theoretical mass (0.41 ppm) of the $[M+Na]^+$ ion of HexSph(d18:1), which is the galacto/glucosyl sphingosine from which the ceramides are formed by addition of a fatty acid moiety. Abbreviations: HexCer, hexosyl ceramide; HexSph, hexosyl sphingosine.

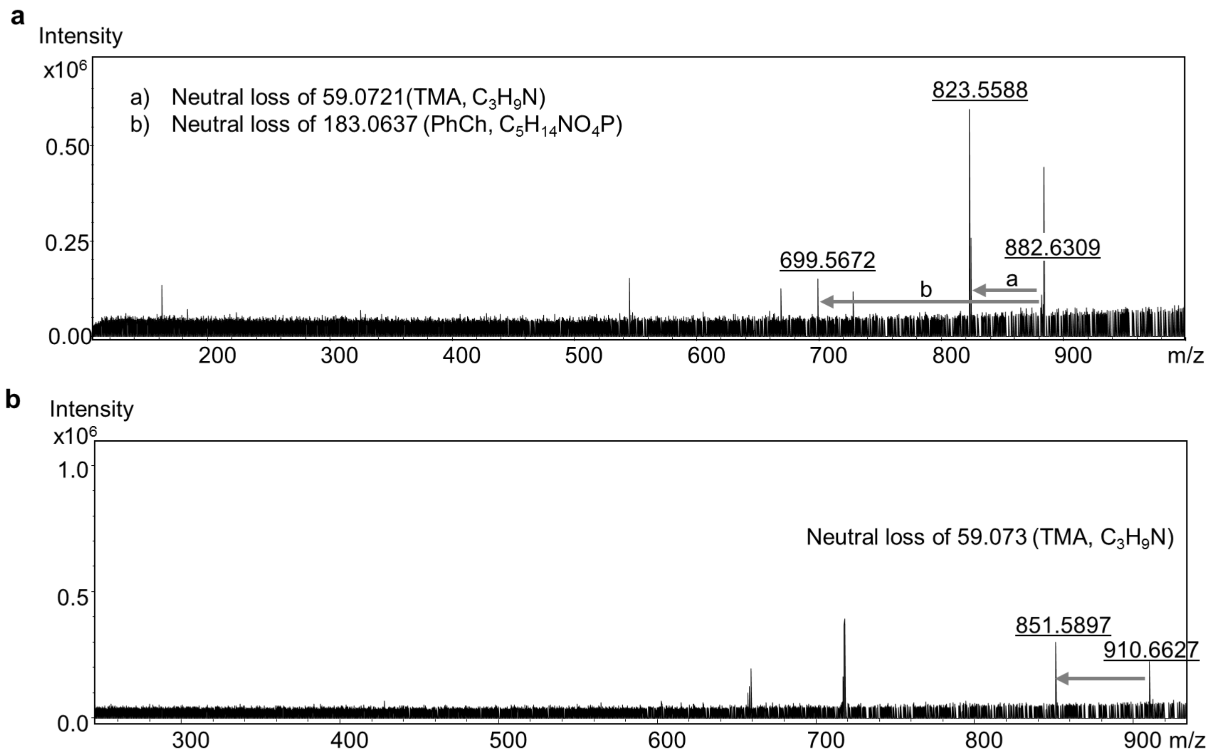

Figure S7. MALDI-MS/MS spectra of PC(t40:1) (*m/z* 882.6309 ([M+K]$^+$), precursor ion) and PC (t42:1) (*m/z* 910.6627 ([M+K]$^+$), precursor ion) in the corpus callosum of mouse brain tissue.

Identification of PC species in tissue by applying MALDI-MS/MSI to isolate the precursor ions **a)** *m/z* 882.63 and **b)** *m/z* 910.65 in a window of 1 Da with collision energy voltage of 20.0- 30.0 V. Abbreviations: TMA, trimethylamine; PhCh, phosphocholine.

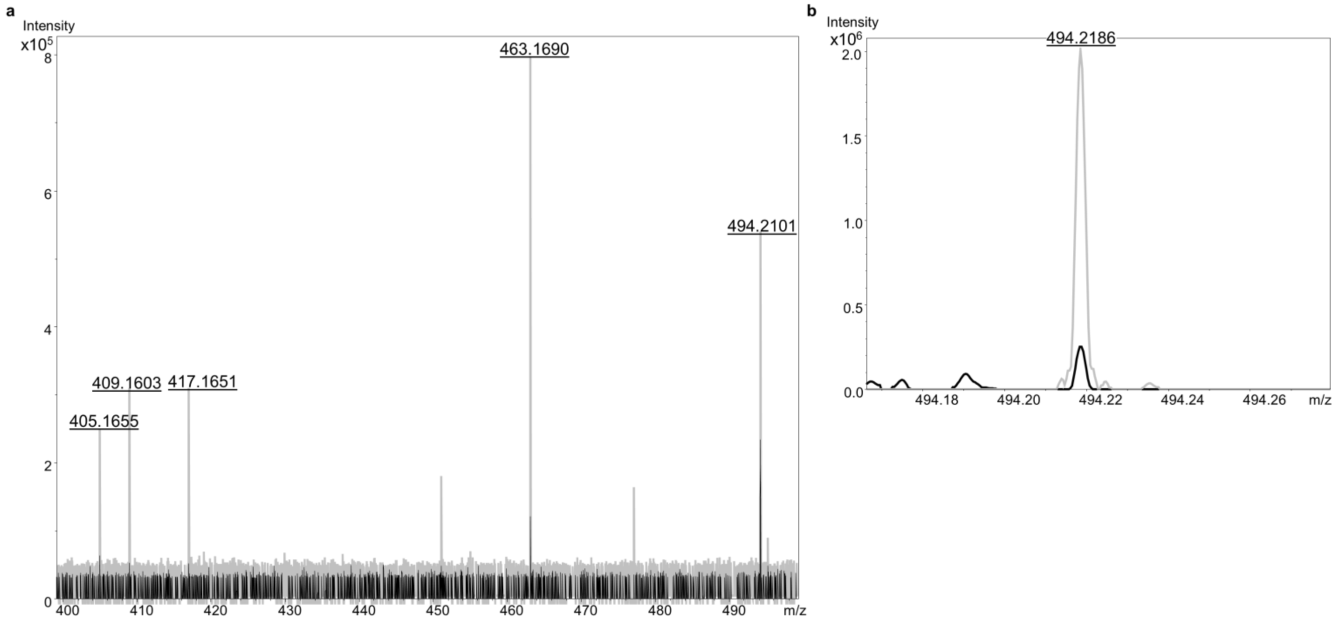

Figure S8. MALDI-MS/MS identification of carnosine.

a) MALDI-MS/MS spectrum of carnosine derivatized by the FMP-10 reactive matrix (*m/z* 494.2101, precursor ion) in the cortex of a mouse brain tissue section (black) and a standard solution (grey). **b)** MALDI-MS spectrum of carnosine derivatized by the FMP-10 reactive matrix (*m/z* 494.21866) in the cortex of a mouse brain tissue section (black) and a standard solution (grey). Although carnosine was detected with application of a regular MALDI matrix, its identification was based on the selective derivatization of the primary amine group by a recently developed MALDI-MSI reactive matrix FMP-10 [3], both in tissue and a standard solution. This approach enabled considerably improved visualization of its brain distribution.

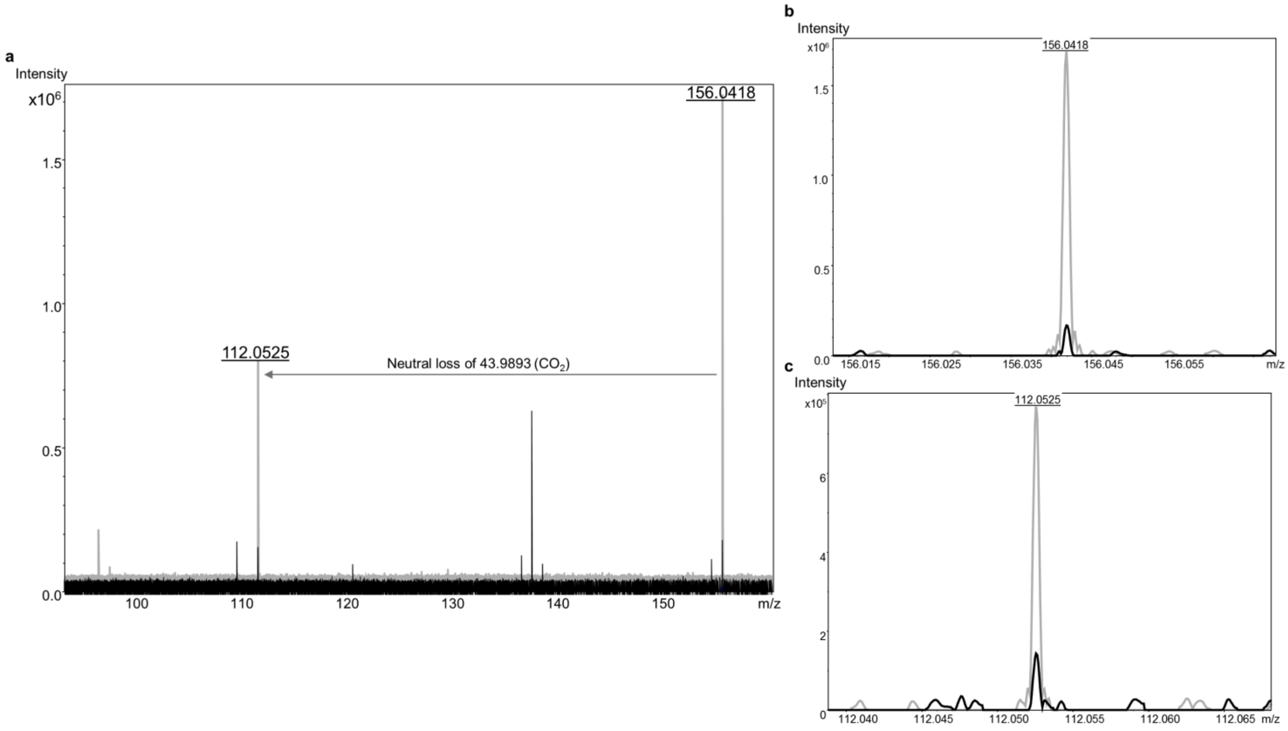

Figure S9. MALDI-MS/MS spectra of betaine (*m/z* 156.0418 ($[M+K]^+$), precursor ion) in the cortex of a mouse brain tissue section (black) and a standard solution (grey).

a) Overlaid overall spectra from the brain tissue (black) and standard solution (grey). **b)** Overlaid spectra from the brain tissue (black) and standard solution (grey) focusing on the precursor ion (*m/z* 156.0418). **c)** Overlaid spectrum from the brain tissue (black) and standard solution (grey) focusing on the main product ion (*m/z* 112.0525). Since only the $[M+K]^+$ adduct was detected in the tissue, phosphate buffer saline at pH 7.4 was added in the standard solution in order to increase its formation. The precursor ion at *m/z* 156.04 was isolated in a window of 1 Da in both the tissue and from standard solution with collision energy voltage of 9.0 V.

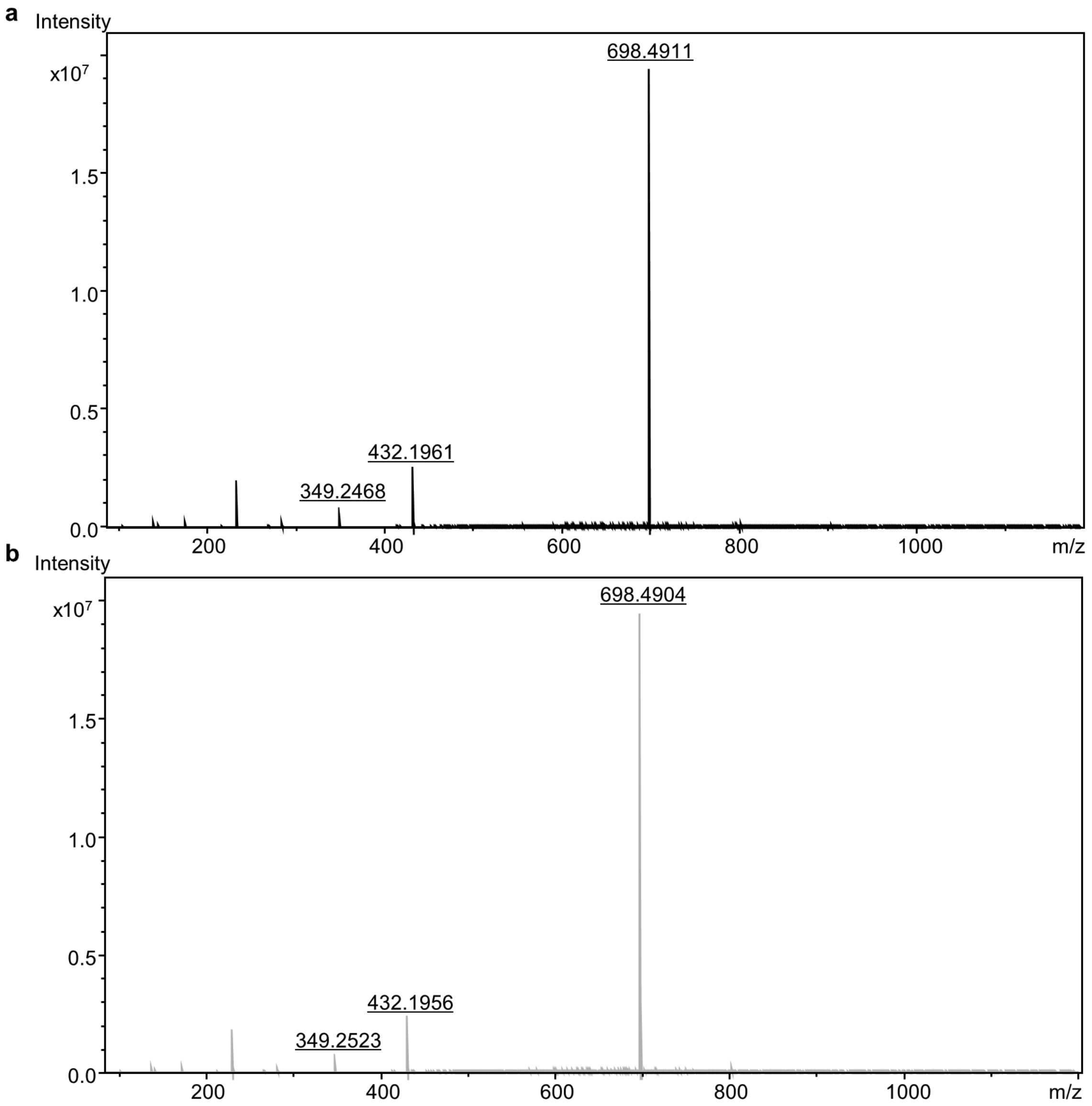

Figure S10. MALDI-MS/MS spectra of α-tocopherol derivatized by the FMP-10 reactive matrix (*m/z* 698.491, precursor ion).

MS/MS spectrum from **a)** cortex of mouse brain tissue (black) and **b)** a standard solution (grey). The precursor ion at *m/z* 698.49 was isolated in a window of 1 Da in both the tissue and standard solution with collision energy voltage of 32.0 V.

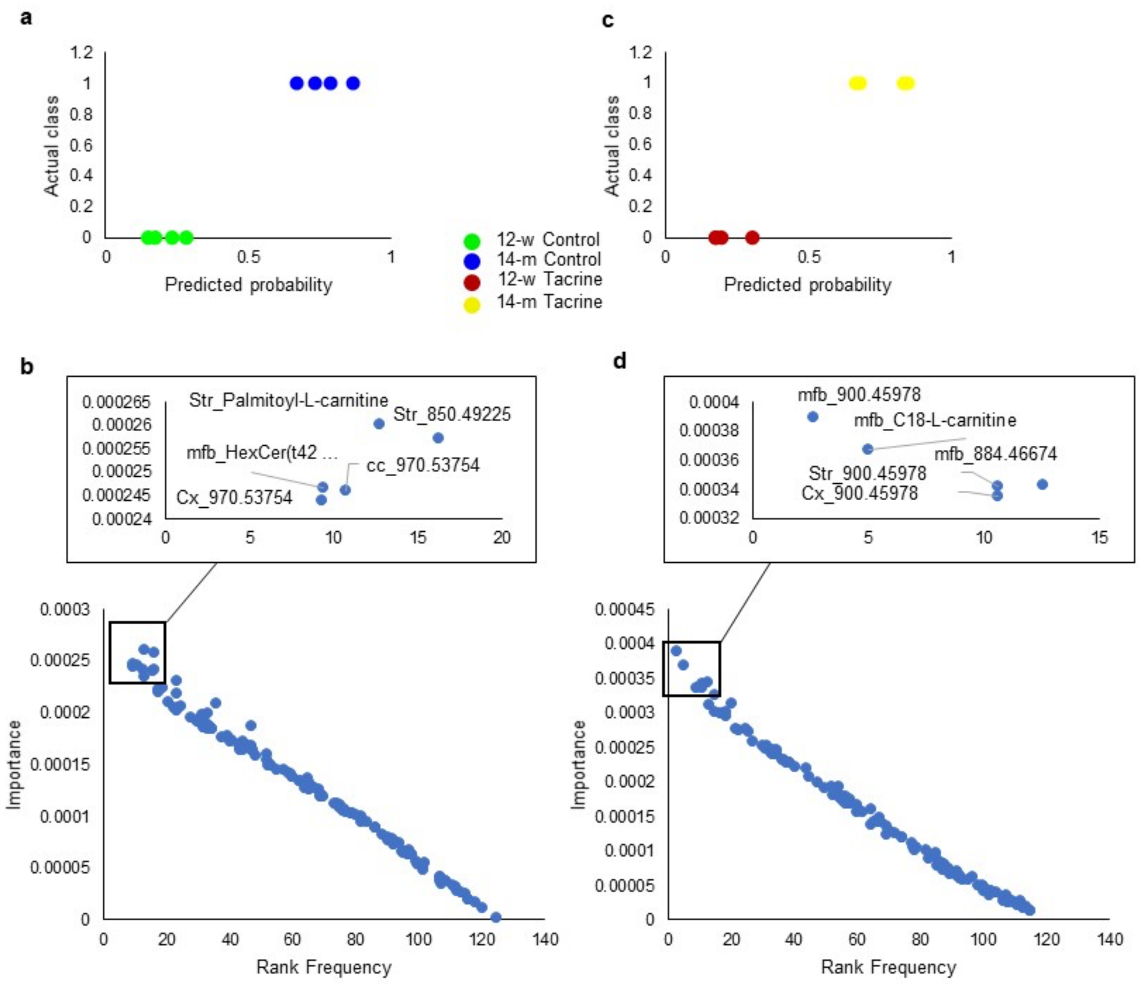

Figure S11. Validation of age-classification performance of significant molecules in examined brain regions by multivariate ROC.

a) Plot of predicted class probabilities for all control samples (0 for 12-w control and 1 for 14-m control). **b**) Plot of the importance of included molecules in the examined brain regions (cc, Cx, mfb, and Str) in the control group, highlighting the five most important ones in a box. **c**) Plot of predicted class probabilities for all tacrine-treated samples (0 for 12-w tacrine and 1 for 14-m tacrine). **d**) Plot of the importance of included molecules in the examined brain region (cc, Cx, mfb, and Str) in the tacrine-treated group, highlighting the five most important ones in a box. Coronal brain level analyzed was 0.26 mm from bregma. Abbreviations: cc, corpus callosum; Cx, cortex; mfb, medial forebrain bundle; Str, striatum.

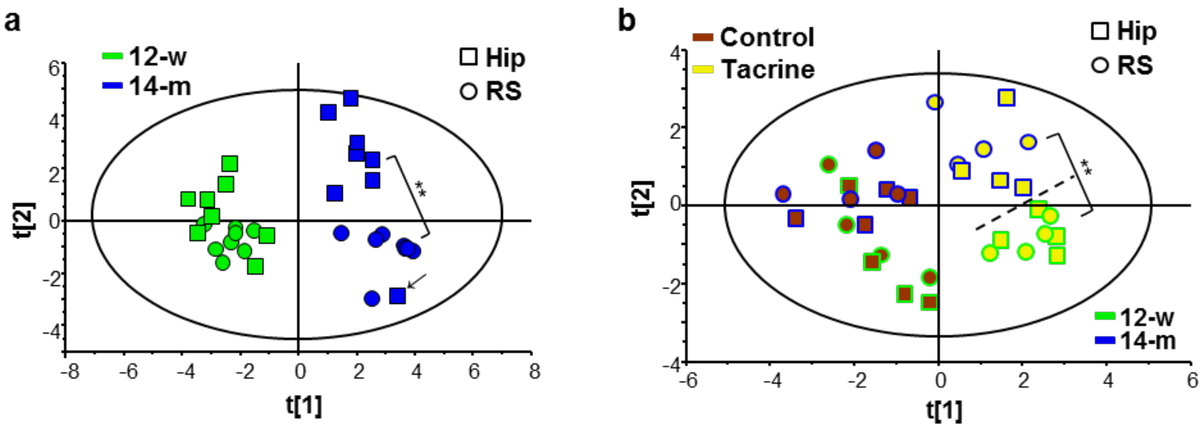

Figure S12. Multivariate analysis of MALDI-MSI data of brain tissue sections at -1.06 mm from bregma.

a) PLS-DA score plot showing the effect of aging on Hip and RS. The 14-m samples are significantly separated according to brain region, despite the presence of one outlier (indicated with a black arrow). **B**) PLS-DA score plot showing the effect of tacrine on Hip and RS. The tacrine-administered samples are significantly separated according to age. Two-way ANOVA with Tukey's post hoc test, ** *P*< 0.01.

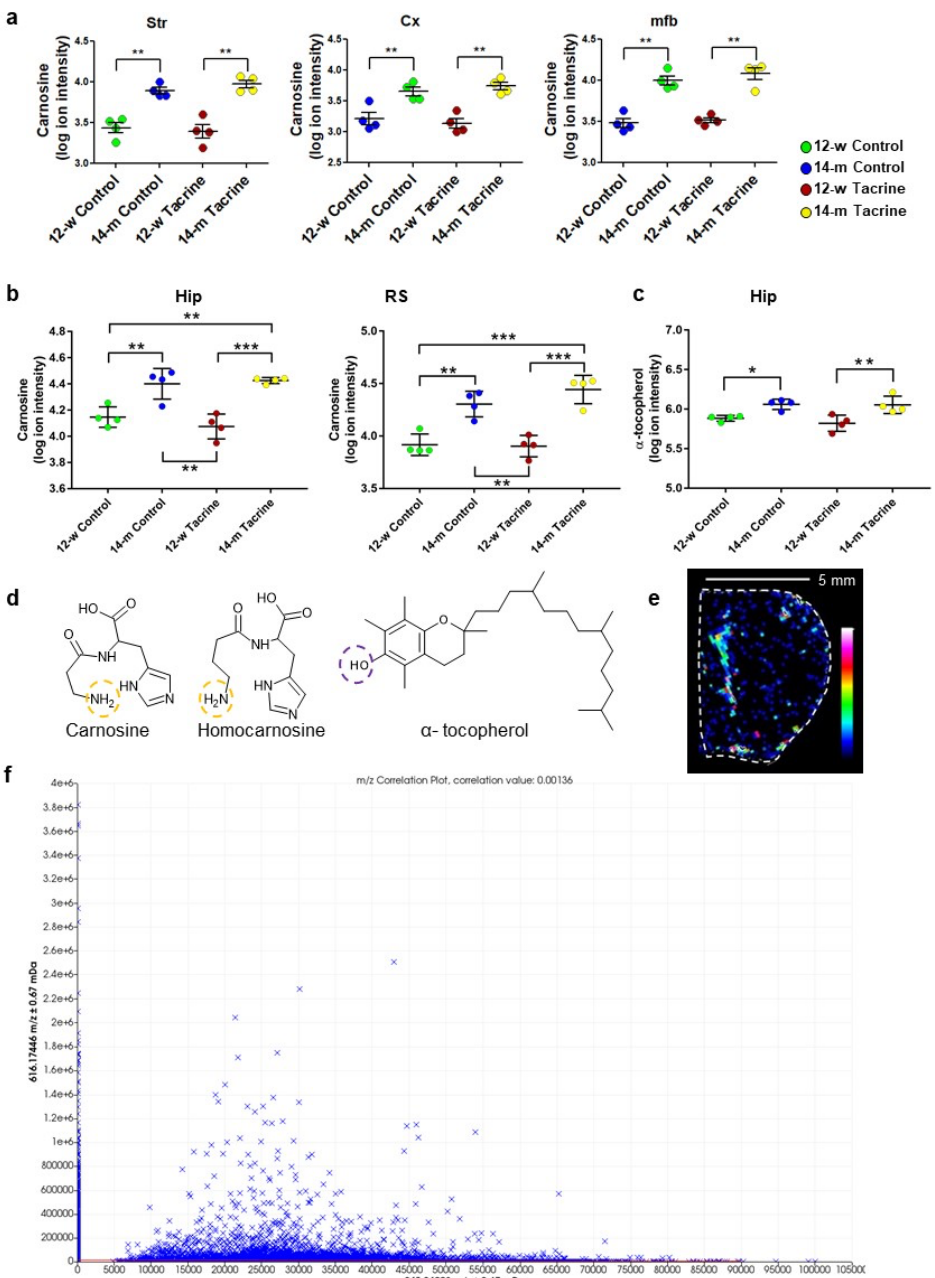

Figure S13. Age-induced alterations of carnosine, homocarnosine, and α-tocopherol.

a) Dot plots of the log ion intensities of carnosine in Str, Cx and mfb (error bars show 95% confidence interval, *n*=4, $^{**}P< 0.01$. **b)** Dot plots of the log ion intensities of carnosine in Hip and RS (error bars show 95% confidence interval, *n*=4, $^{**}P< 0.01$, $^{***}P< 0.001$ **c)** Dot plot of the log ion intensities of α-tocopherol in the hippocampus (*n*=4). Error bars show 95% confidence interval. $^{*}P<0.05$, $^{**}P< 0.01$. **d)** Chemical structures of carnosine , homocarnosine and α-tocopherol showing the primary amines and the phenolic hydroxyl as the sites of FMP-10 derivatization**). e)** MALDI-MS image of the lateral distribution of heme b (*m/z* 616.1175) in a coronal mouse brain tissue section of a 12-w control animal (0.26 mm from bregma). f) Spatial correlation between heme b (*m/z* 616.1175) and carnosine (*m/z* 265.0699) based on their intensity per pixel in a coronal mouse brain tissue section, illustrating a low correlation (r=0.00136). All data are normalized to the RMS.

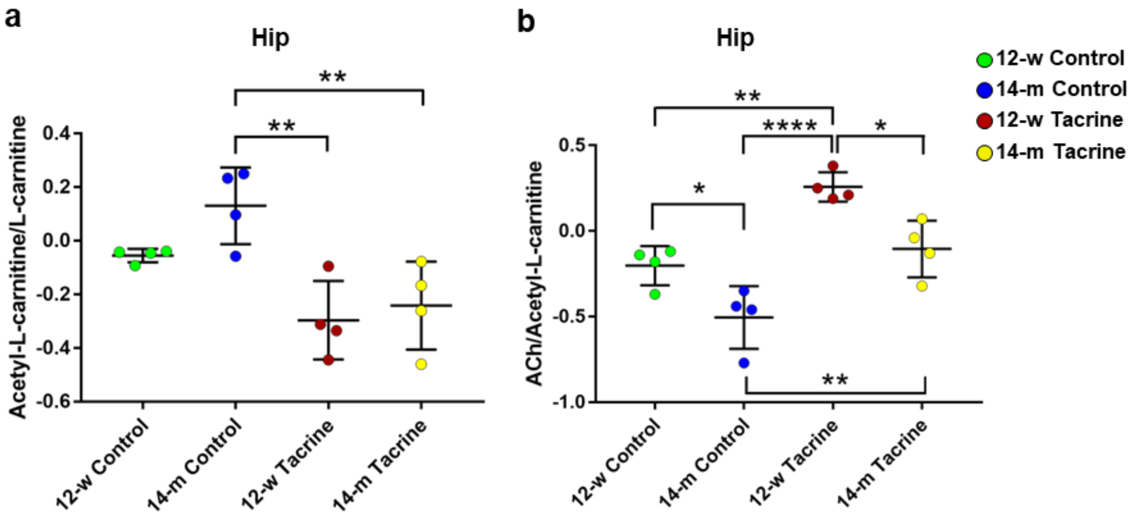

Figure S14. Age and tacrine effects on acetyl-L-carnitine/L-carnitine turnover ratio and ACh/ acetyl-L-carnitine ratio.

a) Dot plot of the log transformed ratio of ion intensities of acetyl-L-carnitine and L-carnitine in the hippocampus (*n*=4), $^{**}P < 0.01$. **b)** Dot plot of the log transformed ratio between ACh ion intensity (normalized to internal standard) and acetyl-L-carnitine ion intensity (normalized to RMS of all data points) in the hippocampus. Error bars show 95% confidence interval (*n*=4), $^{*}P < 0.05$, $^{**}P < 0.01$, $^{***}P < 0.001$, $^{****}P < 0.0001$.

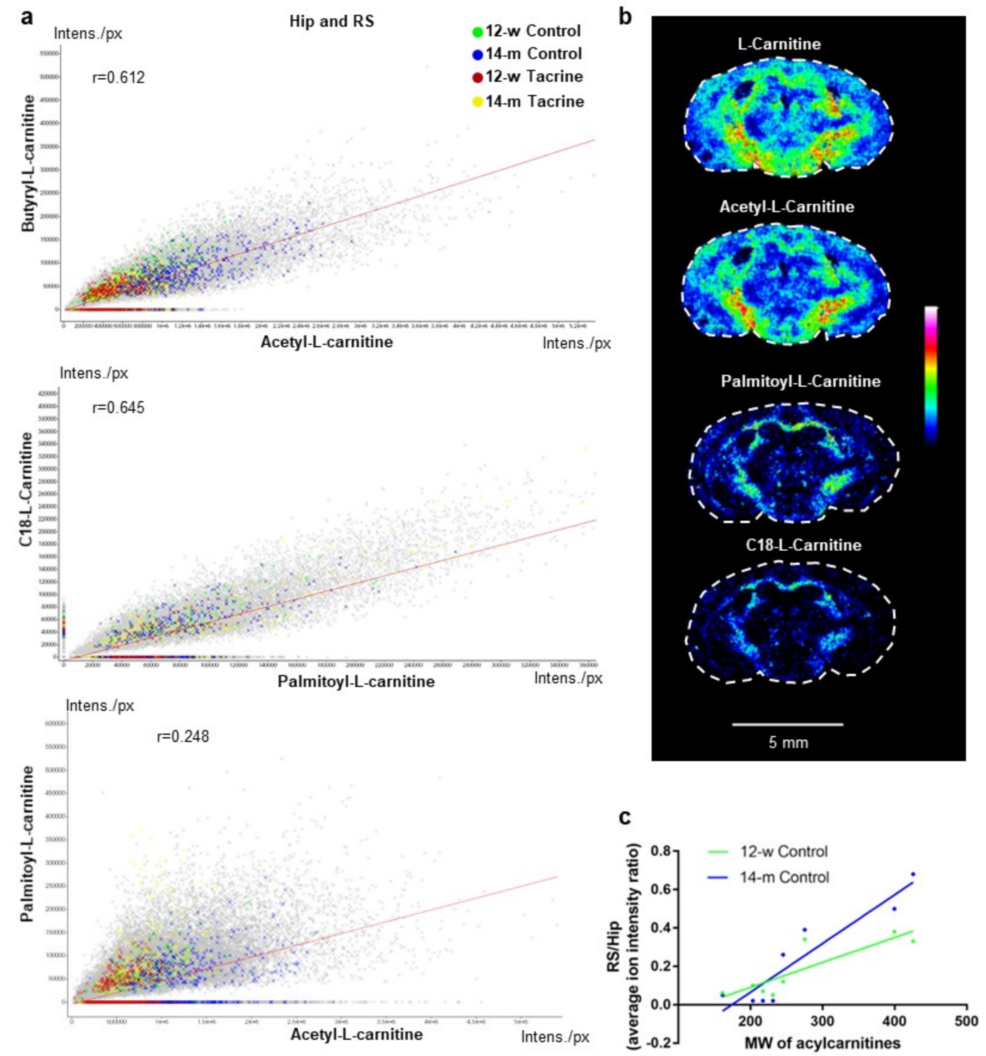

Figure S15. Brain distribution of acylcarnitine species.

a) Spatial correlation between significantly altered (by aging and tacrine) (acyl)-carnitine species based on their intensity per pixel in coronal mouse brain tissue sections. Untargeted spatial co-localization analysis based on Pearson's correlation (*r*) showing a high correlation in the whole brain between acetyl-L-carnitine and butyryl-L-carnitine and between palmitoyl-L-carnitine and C18-L-carnitine. Spatial co-localization of acetyl-L-carnitine and palmitoyl-L-carnitine at the same brain level (-1.06 mm bregma) illustrating a low correlation between short and long fatty acid chain acylcarnitines. Pixels localized in the hippocampus and retrosplenial cortex are highlighted in color, according to the groups. Tissue sections were obtained at -1.06 mm from bregma [4] and the lateral resolution was 100 µm. Abbreviations: 12-w, 12 weeks; 14-m, 14 months; Hip, hippocampus; RS, retrosplenial cortex. **b)** MALDI-MSI of the lateral distribution of L-carnitine (*m/z* 162.1123), acetyl-L-carnitine (*m/z* 204.1226), palmitoyl-L-carnitine (*m/z* 400.3390) and C18-L-carnitine (*m/z* 426.354) in a coronal mouse brain tissue section of a 14-m control animal (-1.06 mm from bregma). The lateral resolution is 80 µm. All data are normalized to the root mean square (RMS) and images are scaled to 100% of maximum intensity. **c)** Impact of the molecular weight of acylcarnitines on their lateral distribution in mouse brain. Linear regression analysis showing significant positive correlation between the molecular weight (MW) of acylcarnitines and their accumulation in the retrosplenial cortex (RS) compared to the hippocampus (Hip) in the 12-w control (R^2=0.739, P<0.01) and 14-m control (R^2=0.858, P<0.01). Abbreviations: 12-w, 12 weeks, 14-m, 14 months; Hip, hippocampus; RS, retrosplenial cortex.

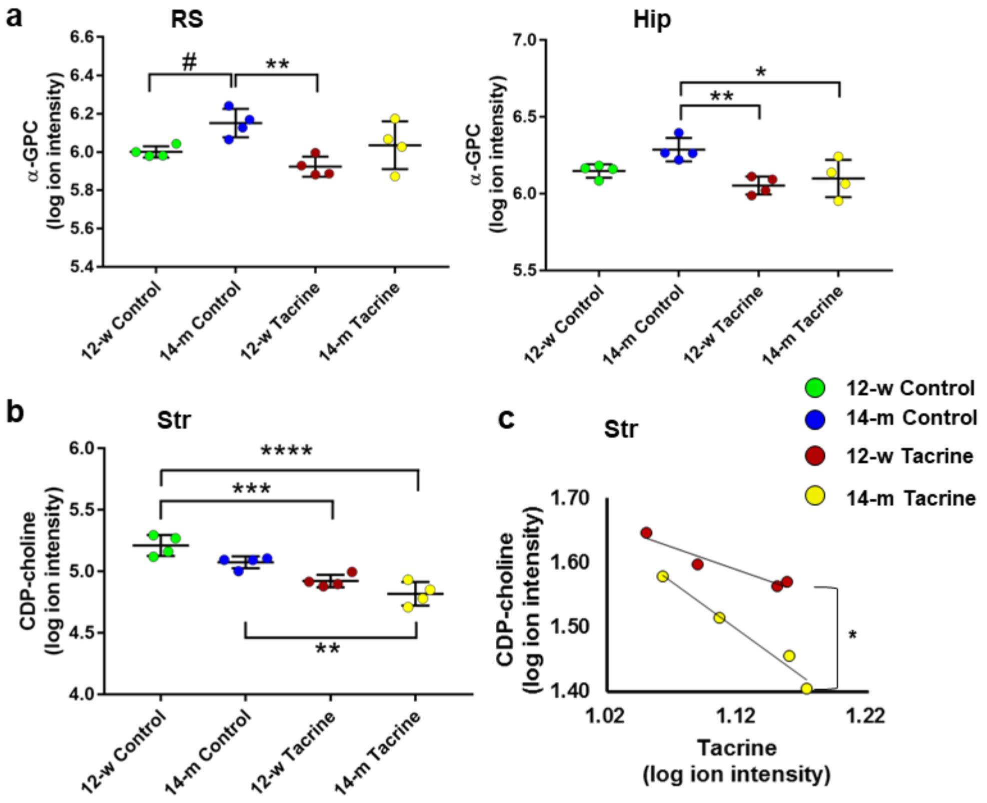

Figure S16. Age and tacrine effects on choline metabolites.

a) Dot plot of the log ion intensity of α-GPC (normalized to RMS) in the Hip and RS of the four different groups at -1.06 mm from bregma. **b**) Dot plot of the log ion intensity of CDP-choline (normalized to RMS) in the Str of the four different groups at bregma 0.26 mm. **c)** Linear correlation between CDP-choline (log ion intensity normalized to RMS) and tacrine (log ion intensity normalized to RMS) in the Str of 12-w (red) and 14-m (yellow) tacrine-dosed animals. The slopes of the two lines are significantly different (*t*-test at α= 0.05 significance level). *P*<0.08, **P*<0.05, ***P*<0.01, ****P*<0.001 and *****P*<0.0001.

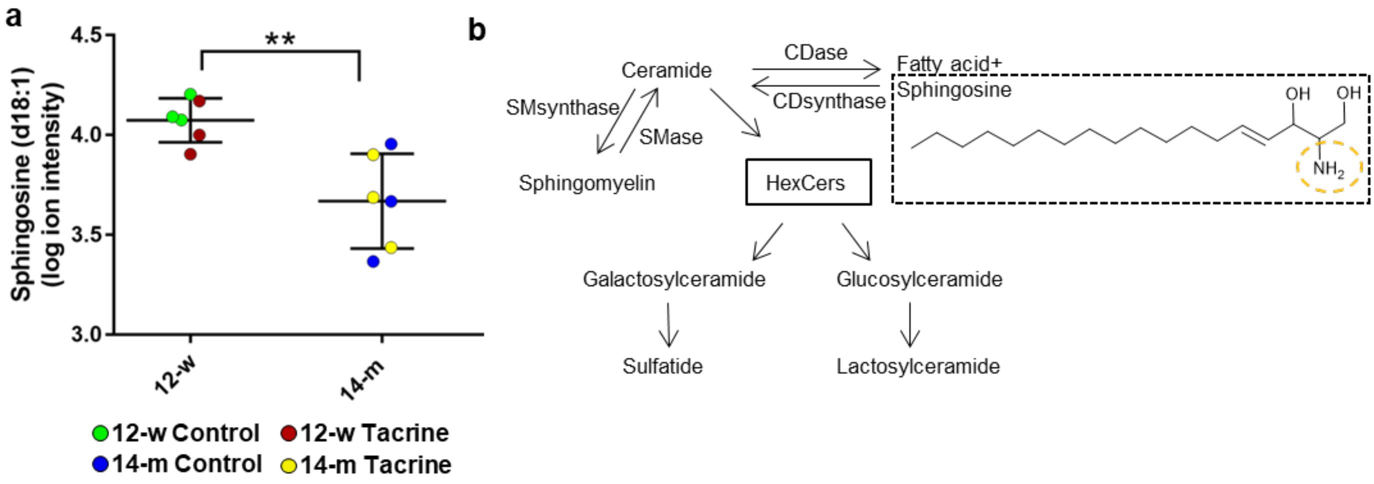

Figure S17. Age and tacrine effects on sphingosine.

a) Dot plot of the log ion intensities of sphingosine(d18:1) extracted from whole sagittal mouse brain tissue sections derivatized with DPP-TFB (*n*=3), ***P*<0.01. Error bars show 95% confidence interval. All data are normalized to the RMS. **b)** Ceramide metabolic pathway. Chemical structure of sphingosine is provided highlighting the DPP-TFB derivatization site.

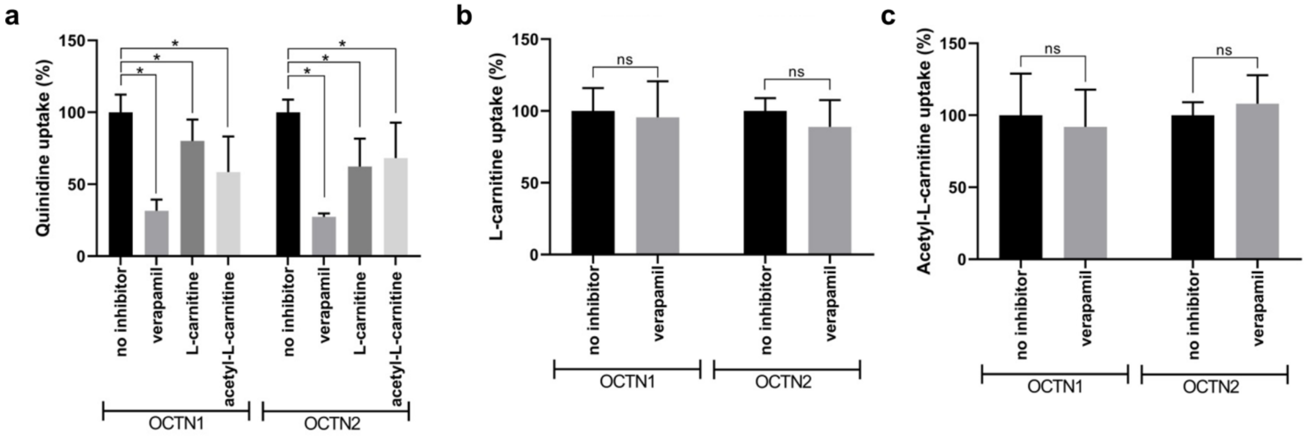

Figure S18. OCTN1 and OCTN2 mediated transport of L-carnitine and acetyl-L-carnitine.

a) Inhibition of OCTN1-and OCTN2-mediated uptake of quinidine by verapamil (model inhibitor), L-carnitine and acetyl-L-carnitine and OCNT1- and OCTN2- mediated uptake of **b)** L-carnitine and **c)** acetyl-L-carnitine. Error bars show 95% confidence interval (*n*=4). *P<0.05. Abbreviations: OCTN, organic cation/carnitine transporter.

Table S1. List of identified metabolites.

The database entry annotation is based on the human metabolome database (www.hmdb.ca) and lipid maps databases (www.lipidmaps.org) for the given mass accuracy. The identification approach is also listed.

Experimental *m/z*	Adduct type	Database entry	Mass accuracy (ppm)	Identification method	Name
146.1176	M^+	HMDB0000895	0.85	Exact mass determination. Brain distribution pattern	Acetylcholine
156.0420	$[M+K]^+$	HMDB0000043	-0.13	MS/MS	Betaine
162.1127	$[M+H]^+$	HMDB0000062	1.23	MS/MS	L-Carnitine
204.1232	$[M+H]^+$	HMDB0000201	0.98	MS/MS	Acetyl-L-carnitine
218.1385	$[M+H]^+$	HMDB0000824<br>HMDB0062514	-0.92	MS/MS	Propionyl-L-carnitine
232.1543	$[M+H]^+$	HMDB0002013<br>HMDB0000736<br>HMDB0062556	-0.08	MS/MS	Butyryl-L-carnitine
248.1486	$[M+H]^+$	HMDB0013127	-2.42	Exact mass determination. Spectrum correlated with other acyl-carnitine species	Hydroxybutyryl-L-carnitine
258.1099	$[M+H]^+$	HMDB0000086	-0.77	MS/MS	α-GPC
265.0696	$[M+K]^+$	HMDB0000033	-0.46	MS/MS on derivatized molecule on tissue. Spectrum compared with standard	Carnosine
279.0851	$[M+K]^+$	HMDB0000745	-0.97	MS/MS on derivatized molecule on tissue. Spectrum compared with carnosine	Homocarnosine
276.1444	$[M+H]^+$	HMDB0013130	0.87	Exact mass determination. Spectrum correlated with other acyl-carnitine species	Glutaryl-L-carnitine

346.2591	$[M+H]^+$	HMDB0061637	0.89	Exact mass determination. Spectrum correlated with other acyl-carnitine species	Hydroxyundecanoyl-L-carnitine
400.3419	$[M+H]^+$	HMDB0000222	-0.50	MS/MS	Palmitoyl-L-carnitine
426.3574	$[M+H]^+$	HMDB0013337 HMDB0013338 HMDB0006351 HMDB0005065 HMDB0094687 HMDB0006464	-0.89	Exact mass determination. Spectrum correlated with other acyl-carnitine species	C-18 L-carnitine
527.0695	$[M+K]^+$	HMDB0001413	-1.98	MS/MS	CDP-choline
836.5960	$[M+K]^+$	LMSP0501AA81	-6.21	Exact mass, distribution pattern and biological effect indicated further unsaturated fatty acid chain of HexCer (t40:1)	HexCer(t40:2)
836.6535	$[M+Na]^+$		-6.10	MS/MS	HexCer(t41:1)
838.6123	$[M+K]^+$		-5.48	MS/MS on $[M+Na]^+$ (*m/z* 822.6397)	HexCer(t40:1)
864.6275	$[M+K]^+$		-5.90	MS/MS on $[M+Na]^+$ (*m/z* 848.6551)	HexCer(t42:2)
882.6290	$[M+K]^+$	LMGP01010659	-6.68	MS/MS	PC(40:1)
910.6604	$[M+K]^+$	LMGP01010826	-6.37	MS/MS	PC(42:1)

Table S2. Pearson correlation coefficient (*r*) between PLS-DA significant metabolites, age and treatment in the Hip and RS.

The red palette indicates positive correlation, whereas the blue palette indicates negative correlation.

Name	Hip		RS	
	Age	Treatment	Age	Treatment
Acetylcholine	-0.188	0.651	-0.401	0.648
Betaine	0.654	0.52	0.385	0.612
L-carnitine	0.654	0.424	0.686	0.424
Acetyl-L-carnitine	0.655	-0.51	0.394	-0.503
α-GPC	0.662	-0.426	0.429	-0.320
Carnosine	0.887	-0.070	0.901	0.122
Homocarnosine	0.592	-0.286	0.779	0.258
OH-decanoyl-L-carnitine	0.373	0.411	0.846	0.176
Palmitoyl-L-carnitine	0.636	0.041	0.704	-0.355
HexCer(t41:1)	0.595	0.207	0.849	0.094
m/z 850.6365	-0.934	0.093	-0.832	0.161
HexCer(t40:1)	0.897	0.061	0.922	-0.038
m/z 856.4822	-0.849	0.366	-0.739	0.104
HexCer(t42:2)	0.749	0.143	0.943	-0.065
m/z 968.5457	-0.757	0.023	-0.459	-0.347
m/z 970.5159	0.648	0.120	0.957	-0.058
m/z 982.5170	0.468	0.288	0.924	0.015

Abbreviations: α-GPC, L-α-glycerophosphocholine; HexCer, hexosyl ceramide.

Table S3. Multiple comparisons with Tukey's post hoc analysis

Metabolite	Column A	Column B	Hip		RS		mfb		Str	
			Mean Dif.*	*P*	Mean Dif.	*P*	Mean Dif.	*P*	Mean Dif.	*P*
ACh	12-w C	14-m C	0.012	0.9993	0.062	0.8919	n.d.	n.d.	n.d.	n.d.
		12-w T	-0.271	0.0744	-0.308	0.0191	n.d.	n.d.	n.d.	n.d.
		14-m T	-0.156	0.4261	-0.087	0.7544	n.d.	n.d.	n.d.	n.d.
	14-m C	12-w C	-0.012	0.9993	-0.062	0.8919	n.d.	n.d.	n.d.	n.d.
		12-w T	-0.283	0.0605	-0.370	0.0056	n.d.	n.d.	n.d.	n.d.
		14-m T	-0.168	0.3658	-0.149	0.3632	n.d.	n.d.	n.d.	n.d.
	12-w T	12-w C	0.271	0.0744	0.308	0.0191	n.d.	n.d.	n.d.	n.d.
		14-m C	0.283	0.0605	0.370	0.0056	n.d.	n.d.	n.d.	n.d.
		14-m T	0.115	0.6622	0.221	0.1062	n.d.	n.d.	n.d.	n.d.
	14-m T	12-w C	0.156	0.4261	0.087	0.7544	n.d.	n.d.	n.d.	n.d.
		14-m C	0.168	0.3658	0.149	0.3632	n.d.	n.d.	n.d.	n.d.
		12-w T	-0.115	0.6622	-0.221	0.1062	n.d.	n.d.	n.d.	n.d.
Betaine	12-w C	14-m C	-0.254	0.0929	-0.141	0.8688	n.d.	n.d.	n.d.	n.d.
		12-w T	-0.195	0.2420	-0.295	0.4146	n.d.	n.d.	n.d.	n.d.
		14-m T	-0.516	0.0009	-0.674	0.0150	n.d.	n.d.	n.d.	n.d.
	14-m C	12-w C	0.254	0.0929	0.141	0.8688	n.d.	n.d.	n.d.	n.d.
		12-w T	0.059	0.9280	-0.154	0.8367	n.d.	n.d.	n.d.	n.d.
		14-m T	-0.262	0.0810	-0.533	0.0569	n.d.	n.d.	n.d.	n.d.
	12-w T	12-w C	0.195	0.2420	0.295	0.4146	n.d.	n.d.	n.d.	n.d.
		14-m C	-0.059	0.9280	0.154	0.8367	n.d.	n.d.	n.d.	n.d.
		14-m T	-0.321	0.0285	-0.379	0.2213	n.d.	n.d.	n.d.	n.d.
	14-m T	12-w C	0.516	0.0009	0.674	0.0150	n.d.	n.d.	n.d.	n.d.
		14-m C	0.262	0.0810	0.533	0.0569	n.d.	n.d.	n.d.	n.d.
		12-w T	0.321	0.0285	0.379	0.2213	n.d.	n.d.	n.d.	n.d.
L-Carnitine	12-w C	14-m C	-0.104	0.2855	-0.093	0.2419	-0.186	0.0856	-0.223	0.0084
		12-w T	-0.052	0.7872	-0.037	0.8534	-0.023	0.9863	-0.037	0.9076
		14-m T	-0.246	0.0039	-0.237	0.0013	-0.141	0.2350	-0.269	0.0020
	14-m C	12-w C	0.104	0.2855	0.093	0.2419	0.186	0.0856	0.223	0.0084
		12-w T	0.053	0.7805	0.056	0.6393	0.162	0.1484	0.186	0.0268
		14-m T	-0.141	0.1012	-0.144	0.0414	0.044	0.9184	-0.046	0.8412
	12-w T	12-w C	0.052	0.7872	0.037	0.8534	0.023	0.9863	0.037	0.9076
		14-m C	-0.053	0.7805	-0.056	0.6393	-0.162	0.1484	-0.186	0.0268
		14-m T	-0.194	0.0198	-0.200	0.0051	-0.118	0.3740	-0.232	0.0063

	14-m T	12-w C	0.246	0.0039	0.237	0.0013	0.141	0.2350	0.269	0.0020
		14-m C	0.141	0.1012	0.144	0.0414	-0.044	0.9184	0.046	0.8412
		12-w T	0.194	0.0198	0.200	0.0051	0.118	0.3740	0.232	0.0063
Acetyl-L-carnitine	12-w C	14-m C	-0.290	0.0390	-0.211	0.3328	-0.157	0.1585	-0.317	0.0089
		12-w T	0.190	0.2290	0.137	0.6694	0.176	0.1028	-0.003	1.0000
		14-m T	-0.060	0.9174	0.042	0.9840	0.181	0.0901	-0.120	0.4694
	14-m C	12-w C	0.290	0.0390	0.211	0.3328	0.157	0.1585	0.317	0.0089
		12-w T	0.479	0.0012	0.348	0.0547	0.333	0.0020	0.315	0.0094
		14-m T	0.230	0.1154	0.253	0.2002	0.339	0.0018	0.197	0.1176
	12-w T	12-w C	-0.190	0.2290	-0.137	0.6694	-0.176	0.1028	0.003	1.0000
		14-m C	-0.479	0.0012	-0.348	0.0547	-0.333	0.0020	-0.315	0.0094
		14-m T	-0.249	0.0827	-0.094	0.8566	0.005	0.9998	-0.117	0.4877
	14-m T	12-w C	0.060	0.9174	-0.042	0.9840	-0.181	0.0901	0.120	0.4694
		14-m C	-0.230	0.1154	-0.253	0.2002	-0.339	0.0018	-0.197	0.1176
		12-w T	0.249	0.0827	0.094	0.8566	-0.005	0.9998	0.117	0.4877
Propionyl-L-carnitine	12-w C	14-m C	-0.145	0.6078	-0.093	0.9159	-0.084	0.8358	-0.187	0.2853
		12-w T	0.398	0.0226	0.326	0.1647	0.219	0.1862	0.221	0.1716
		14-m T	0.042	0.9836	-0.025	0.9982	0.167	0.3838	0.040	0.9778
	14-m C	12-w C	0.145	0.6078	0.093	0.9159	0.084	0.8358	0.187	0.2853
		12-w T	0.543	0.0026	0.419	0.0568	0.303	0.0466	0.408	0.0069
		14-m T	0.187	0.4094	0.069	0.9634	0.251	0.1107	0.227	0.1574
	12-w T	12-w C	-0.398	0.0226	-0.326	0.1647	-0.219	0.1862	-0.221	0.1716
		14-m C	-0.543	0.0026	-0.419	0.0568	-0.303	0.0466	-0.408	0.0069
		14-m T	-0.356	0.0422	-0.351	0.1257	-0.051	0.9549	-0.182	0.3081
	14-m T	12-w C	-0.042	0.9836	0.025	0.9982	-0.167	0.3838	-0.040	0.9778
		14-m C	-0.187	0.4094	-0.069	0.9634	-0.251	0.1107	-0.227	0.1574
		12-w T	0.356	0.0422	0.351	0.1257	0.051	0.9549	0.182	0.3081
Butyryl-L-carnitine	12-w C	14-m C	-0.217	0.1989	-0.187	0.4318	-0.018	0.9949	-0.126	0.4719
		12-w T	0.278	0.0744	0.473	0.0087	0.311	0.0073	0.280	0.0274
		14-m T	-0.035	0.9853	0.057	0.9619	0.388	0.0013	0.192	0.1583
	14-m C	12-w C	0.217	0.1989	0.187	0.4318	0.018	0.9949	0.126	0.4719
		12-w T	0.495	0.0019	0.660	0.0006	0.329	0.0048	0.406	0.0021
		14-m T	0.182	0.3263	0.244	0.2248	0.406	0.0009	0.318	0.0124
	12-w T	12-w C	-0.278	0.0744	-0.473	0.0087	-0.311	0.0073	-0.280	0.0274
		14-m C	-0.495	0.0019	-0.660	0.0006	-0.329	0.0048	-0.406	0.0021

		14-m T	-0.313	0.0412	-0.416	0.0203	0.077	0.7484	-0.088	0.7312
	14-m T	12-w C	0.035	0.9853	-0.057	0.9619	-0.388	0.0013	-0.192	0.1583
		14-m C	-0.182	0.3263	-0.244	0.2248	-0.406	0.0009	-0.318	0.0124
		12-w T	0.313	0.0412	0.416	0.0203	-0.077	0.7484	0.088	0.7312
Hydroxybutyryl-L-carnitine	12-w C	14-m C	0.147	0.8883	0.013	0.9999	n.d.	n.d.	n.d.	n.d.
		12-w T	0.524	0.1009	0.941	0.0097	n.d.	n.d.	n.d.	n.d.
		14-m T	0.360	0.3395	0.424	0.2666	n.d.	n.d.	n.d.	n.d.
	14-m C	12-w C	-0.147	0.8883	-0.013	0.9999	n.d.	n.d.	n.d.	n.d.
		12-w T	0.377	0.3040	0.928	0.0107	n.d.	n.d.	n.d.	n.d.
		14-m T	0.213	0.7312	0.411	0.2891	n.d.	n.d.	n.d.	n.d.
	12-w T	12-w C	-0.524	0.1009	-0.941	0.0097	n.d.	n.d.	n.d.	n.d.
		14-m C	-0.377	0.3040	-0.928	0.0107	n.d.	n.d.	n.d.	n.d.
		14-m T	-0.164	0.8536	-0.517	0.1851	n.d.	n.d.	n.d.	n.d.
	14-m T	12-w C	-0.360	0.3395	-0.424	0.2666	n.d.	n.d.	n.d.	n.d.
		14-m C	-0.213	0.7312	-0.411	0.2891	n.d.	n.d.	n.d.	n.d.
		12-w T	0.164	0.8536	0.517	0.1851	n.d.	n.d.	n.d.	n.d.
alpha GPC	12-w C	14-m C	-0.150	0.0793	-0.139	0.1227	-0.079	0.5943	-0.145	0.2684
		12-w T	0.077	0.5288	0.095	0.3841	0.122	0.2527	0.048	0.9182
		14-m T	-0.035	0.9223	0.049	0.8272	0.159	0.0974	0.070	0.7891
	14-m C	12-w C	0.150	0.0793	0.139	0.1227	0.079	0.5943	0.145	0.2684
		12-w T	0.227	0.0071	0.233	0.0070	0.200	0.0310	0.193	0.0996
		14-m T	0.115	0.2158	0.187	0.0285	0.238	0.0107	0.216	0.0606
	12-w T	12-w C	-0.077	0.5288	-0.095	0.3841	-0.122	0.2527	-0.048	0.9182
		14-m C	-0.227	0.0071	-0.233	0.0070	-0.200	0.0310	-0.193	0.0996
		14-m T	-0.112	0.2365	-0.046	0.8506	0.038	0.9274	0.022	0.9906
	14-m T	12-w C	0.035	0.9223	-0.049	0.8272	-0.159	0.0974	-0.070	0.7891
		14-m C	-0.115	0.2158	-0.187	0.0285	-0.238	0.0107	-0.216	0.0606
		12-w T	0.112	0.2365	0.046	0.8506	-0.038	0.9274	-0.022	0.9906
Carnosine	12-w C	14-m C	-0.254	0.0059	-0.388	0.0024	-0.515	0.0001	-0.453	0.0009
		12-w T	0.072	0.6404	0.013	0.9986	-0.032	0.9742	0.045	0.9518
		14-m T	-0.279	0.0029	-0.527	0.0002	-0.599	0.0000	-0.535	0.0002
	14-m C	12-w C	0.254	0.0059	0.388	0.0024	0.515	0.0001	0.453	0.0009
		12-w T	0.326	0.0008	0.401	0.0018	0.483	0.0002	0.498	0.0004
		14-m T	-0.025	0.9753	-0.138	0.3724	-0.084	0.6975	-0.083	0.7700
	12-w T	12-w C	-0.072	0.6404	-0.013	0.9986	0.032	0.9742	-0.045	0.9518

		14-m C	-0.326	0.0008	-0.401	0.0018	-0.483	0.0002	-0.498	0.0004
		14-m T	-0.351	0.0004	-0.539	0.0001	-0.567	0.0000	-0.580	0.0001
	14-m T	12-w C	0.279	0.0029	0.527	0.0002	0.599	0.0000	0.535	0.0002
		14-m C	0.025	0.9753	0.138	0.3724	0.084	0.6975	0.083	0.7700
		12-w T	0.351	0.0004	0.539	0.0001	0.567	0.0000	0.580	0.0001
Glutaryl-L-carnitine	12-w C	14-m C	0.043	0.9921	-0.008	0.9999	-0.032	0.9255	-0.028	0.9382
		12-w T	0.398	0.0950	0.307	0.1063	0.199	0.0112	0.164	0.0247
		14-m T	0.021	0.9991	0.020	0.9984	0.088	0.3663	0.076	0.4378
	14-m C	12-w C	-0.043	0.9921	0.008	0.9999	0.032	0.9255	0.028	0.9382
		12-w T	0.355	0.1494	0.315	0.0954	0.231	0.0039	0.192	0.0091
		14-m T	-0.022	0.9989	0.028	0.9956	0.120	0.1496	0.104	0.1992
	12-w T	12-w C	-0.398	0.0950	-0.307	0.1063	-0.199	0.0112	-0.164	0.0247
		14-m C	-0.355	0.1494	-0.315	0.0954	-0.231	0.0039	-0.192	0.0091
		14-m T	-0.377	0.1187	-0.287	0.1386	-0.110	0.1998	-0.089	0.3120
	14-m T	12-w C	-0.021	0.9991	-0.020	0.9984	-0.088	0.3663	-0.076	0.4378
		14-m C	0.022	0.9989	-0.028	0.9956	-0.120	0.1496	-0.104	0.1992
		12-w T	0.377	0.1187	0.287	0.1386	0.110	0.1998	0.089	0.3120
Homocarnosine	12-w C	14-m C	-0.133	0.1881	-0.175	0.0668	-0.467	0.0028	-0.352	0.0136
		12-w T	0.043	0.8947	-0.031	0.9592	-0.106	0.7246	-0.019	0.9970
		14-m T	-0.061	0.7519	-0.286	0.0030	-0.473	0.0025	-0.403	0.0053
	14-m C	12-w C	0.133	0.1881	0.175	0.0668	0.467	0.0028	0.352	0.0136
		12-w T	0.176	0.0596	0.144	0.1508	0.361	0.0169	0.333	0.0194
		14-m T	0.071	0.6587	-0.112	0.3236	-0.006	0.9999	-0.052	0.9458
	12-w T	12-w C	-0.043	0.8947	0.031	0.9592	0.106	0.7246	0.019	0.9970
		14-m C	-0.176	0.0596	-0.144	0.1508	-0.361	0.0169	-0.333	0.0194
		14-m T	-0.104	0.3643	-0.256	0.0070	-0.368	0.0151	-0.384	0.0075
	14-m T	12-w C	0.061	0.7519	0.286	0.0030	0.473	0.0025	0.403	0.0053
		14-m C	-0.071	0.6587	0.112	0.3236	0.006	0.9999	0.052	0.9458
		12-w T	0.104	0.3643	0.256	0.0070	0.368	0.0151	0.384	0.0075
Hydroxydecanoyl-L-carnitine	12-w C	14-m C	-0.020	0.9997	-0.728	0.0073	-0.490	0.0085	-0.637	0.0007
		12-w T	-0.048	0.9961	-0.145	0.8679	-0.058	0.9643	-0.107	0.7936
		14-m T	-0.554	0.1042	-0.781	0.0045	-0.465	0.0121	-0.643	0.0006
	14-m C	12-w C	0.020	0.9997	0.728	0.0073	0.490	0.0085	0.637	0.0007
		12-w T	-0.027	0.9993	0.584	0.0436	0.432	0.0193	0.530	0.0030
		14-m T	-0.533	0.1214	-0.052	0.9902	0.025	0.9968	-0.006	0.9999

	12-w T	12-w C	0.048	0.9961	0.145	0.8679	0.058	0.9643	0.107	0.7936
		14-m C	0.027	0.9993	-0.584	0.0436	-0.432	0.0193	-0.530	0.0030
		14-m T	-0.506	0.1484	-0.636	0.0274	-0.407	0.0276	-0.536	0.0027
	14-m T	12-w C	0.554	0.1042	0.781	0.0045	0.465	0.0121	0.643	0.0006
		14-m C	0.533	0.1214	0.052	0.9902	-0.025	0.9968	0.006	0.9999
		12-w T	0.506	0.1484	0.636	0.0274	0.407	0.0276	0.536	0.0027
Palmitoyl-L-carnitine	12-w C	14-m C	-0.186	0.7093	-0.306	0.0407	0.005	0.9998	-0.170	0.0377
		12-w T	0.176	0.7425	0.114	0.6664	0.209	0.0364	0.024	0.9692
		14-m T	-0.412	0.1338	-0.139	0.5247	0.209	0.0363	-0.073	0.5495
	14-m C	12-w C	0.186	0.7093	0.306	0.0407	-0.005	0.9998	0.170	0.0377
		12-w T	0.363	0.2095	0.420	0.0055	0.204	0.0419	0.194	0.0173
		14-m T	-0.226	0.5771	0.168	0.3694	0.204	0.0418	0.097	0.3290
	12-w T	12-w C	-0.176	0.7425	-0.114	0.6664	-0.209	0.0364	-0.024	0.9692
		14-m C	-0.363	0.2095	-0.420	0.0055	-0.204	0.0419	-0.194	0.0173
		14-m T	-0.589	0.0236	-0.253	0.1020	0.000	1.0000	-0.098	0.3205
	14-m T	12-w C	0.412	0.1338	0.139	0.5247	-0.209	0.0363	0.073	0.5495
		14-m C	0.226	0.5771	-0.168	0.3694	-0.204	0.0418	-0.097	0.3290
		12-w T	0.589	0.0236	0.253	0.1020	0.000	1.0000	0.098	0.3205
C18-L-carnitine	12-w C	14-m C	-0.029	0.9993	-0.377	0.2239	0.021	0.9861	-0.140	0.1960
		12-w T	0.165	0.8976	0.266	0.4961	0.533	0.0000	0.144	0.1773
		14-m T	-0.331	0.5273	-0.105	0.9392	0.390	0.0002	0.043	0.9117
	14-m C	12-w C	0.029	0.9993	0.377	0.2239	-0.021	0.9861	0.140	0.1960
		12-w T	0.194	0.8466	0.643	0.0199	0.512	0.0000	0.284	0.0046
		14-m T	-0.302	0.5969	0.272	0.4778	0.369	0.0004	0.183	0.0675
	12-w T	12-w C	-0.165	0.8976	-0.266	0.4961	-0.533	0.0000	-0.144	0.1773
		14-m C	-0.194	0.8466	-0.643	0.0199	-0.512	0.0000	-0.284	0.0046
		14-m T	-0.496	0.2125	-0.371	0.2351	-0.143	0.1596	-0.101	0.4411
	14-m T	12-w C	0.331	0.5273	0.105	0.9392	-0.390	0.0002	-0.043	0.9117
		14-m C	0.302	0.5969	-0.272	0.4778	-0.369	0.0004	-0.183	0.0675
		12-w T	0.496	0.2125	0.371	0.2351	0.143	0.1596	0.101	0.4411
CDP-choline	12-w C	14-m C	-0.086	0.8351	0.001	1.0000	0.031	0.9874	0.1208	0.0628
		12-w T	0.810	0.0000	0.221	0.0459	0.359	0.0119	0.2530	0.0003
		14-m T	0.612	0.0003	0.385	0.0010	0.347	0.0149	0.3584	<0.0001
	14-m C	12-w C	0.086	0.8351	-0.001	1.0000	-0.031	0.9874	-0.1208	0.0628

		12-w T	0.896	0.0000	0.220	0.0473	0.328	0.0211	0.1322	0.0396
		14-m T	0.698	0.0001	0.384	0.0010	0.316	0.0264	0.2376	0.0006
	12-w T	12-w C	-0.810	0.0000	-0.221	0.0459	-0.359	0.0119	-0.2530	0.0003
		14-m C	-0.896	0.0000	-0.220	0.0473	-0.328	0.0211	-0.1322	0.0396
		14-m T	-0.198	0.2643	0.164	0.1701	-0.012	0.9992	0.1055	0.1149
	14-m T	12-w C	-0.612	0.0003	-0.385	0.0010	-0.347	0.0149	-0.3584	<0.0001
		14-m C	-0.698	0.0001	-0.384	0.0010	-0.316	0.0264	-0.2376	0.0006
		12-w T	0.198	0.2643	-0.164	0.1701	0.012	0.9992	-0.1055	0.1149
HexCer(t40:2)	12-w C	14-m C	-0.425	0.6741	n.c	n.c	-0.518	0.0053	-1.020	0.0000
		12-w T	-0.239	0.9023	n.c	n.c	0.132	0.7025	-0.103	0.7937
		14-m T	-1.050	0.0847	n.c	n.c	-0.478	0.0092	-1.001	0.0000
	14-m C	12-w C	0.425	0.6741	n.c	n.c	0.518	0.0053	1.020	0.0000
		12-w T	0.186	0.9293	n.c	n.c	0.650	0.0009	0.917	0.0000
		14-m T	-0.624	0.3070	n.c	n.c	0.039	0.9875	0.019	0.9982
	12-w T	12-w C	0.239	0.9023	n.c	n.c	-0.132	0.7025	0.103	0.7937
		14-m C	-0.186	0.9293	n.c	n.c	-0.650	0.0009	-0.917	0.0000
		14-m T	-0.811	0.1151	n.c	n.c	-0.610	0.0015	-0.898	0.0000
	14-m T	12-w C	1.050	0.0847	n.c	n.c	0.478	0.0092	1.001	0.0000
		14-m C	0.624	0.3070	n.c	n.c	-0.039	0.9875	-0.019	0.9982
		12-w T	0.811	0.1151	n.c	n.c	0.610	0.0015	0.898	0.0000
HexCer(t40:1)	12-w C	14-m C	-0.231	0.3569	-0.309	0.0107	-0.181	0.0161	-0.249	0.0113
		12-w T	-0.066	0.9594	-0.024	0.9907	-0.021	0.9744	-0.087	0.5631
		14-m T	-0.340	0.1039	-0.357	0.0038	-0.132	0.0866	-0.181	0.0690
	14-m C	12-w C	0.231	0.3569	0.309	0.0107	0.181	0.0161	0.249	0.0113
		12-w T	0.165	0.6231	0.285	0.0179	0.160	0.0334	0.163	0.1121
		14-m T	-0.110	0.8443	-0.048	0.9313	0.049	0.7671	0.068	0.7304
	12-w T	12-w C	0.066	0.9594	0.024	0.9907	0.021	0.9744	0.087	0.5631
		14-m C	-0.165	0.6231	-0.285	0.0179	-0.160	0.0334	-0.163	0.1121
		14-m T	-0.275	0.2255	-0.333	0.0063	-0.111	0.1705	-0.095	0.4938
	14-m T	12-w C	0.340	0.1039	0.357	0.0038	0.132	0.0866	0.181	0.0690
		14-m C	0.110	0.8443	0.048	0.9313	-0.049	0.7671	-0.068	0.7304
		12-w T	0.275	0.2255	0.333	0.0063	0.111	0.1705	0.095	0.4938
HexCer(t41:1)	12-w C	14-m C	-0.418	0.0013	-0.432	0.0008	-0.570	0.0001	-1.068	0.0002
		12-w T	-0.032	0.9798	0.071	0.8090	0.076	0.8060	-0.026	0.9987
		14-m T	-0.442	0.0008	-0.463	0.0004	-0.504	0.0004	-1.037	0.0002
	14-m C	12-w C	0.418	0.0013	0.432	0.0008	0.570	0.0001	1.068	0.0002

		12-w T	0.386	0.0025	0.503	0.0002	0.647	0.0000	1.043	0.0002
		14-m T	-0.024	0.9905	-0.032	0.9774	0.066	0.8654	0.031	0.9977
	12-w T	12-w C	0.032	0.9798	-0.071	0.8090	-0.076	0.8060	0.026	0.9987
		14-m C	-0.386	0.0025	-0.503	0.0002	-0.647	0.0000	-1.043	0.0002
		14-m T	-0.410	0.0016	-0.535	0.0001	-0.581	0.0001	-1.012	0.0003
	14-m T	12-w C	0.442	0.0008	0.463	0.0004	0.504	0.0004	1.037	0.0002
		14-m C	0.024	0.9905	0.032	0.9774	-0.066	0.8654	-0.031	0.9977
		12-w T	0.410	0.0016	0.535	0.0001	0.581	0.0001	1.012	0.0003
HexCer(t42:2)	12-w C	14-m C	-0.255	0.1157	-0.357	0.0001	-0.287	0.0015	-0.379	0.0003
		12-w T	-0.015	0.9989	0.039	0.8798	0.014	0.9947	-0.071	0.6677
		14-m T	-0.354	0.0226	-0.345	0.0001	-0.269	0.0025	-0.370	0.0003
	14-m C	12-w C	0.255	0.1157	0.357	0.0001	0.287	0.0015	0.379	0.0003
		12-w T	0.240	0.1458	0.396	0.0000	0.301	0.0010	0.308	0.0016
		14-m T	-0.098	0.7771	0.013	0.9949	0.018	0.9895	0.009	0.9989
	12-w T	12-w C	0.015	0.9989	-0.039	0.8798	-0.014	0.9947	0.071	0.6677
		14-m C	-0.240	0.1458	-0.396	0.0000	-0.301	0.0010	-0.308	0.0016
		14-m T	-0.339	0.0290	-0.383	0.0000	-0.283	0.0017	-0.299	0.0020
	14-m T	12-w C	0.354	0.0226	0.345	0.0001	0.269	0.0025	0.370	0.0003
		14-m C	0.098	0.7771	-0.013	0.9949	-0.018	0.9895	-0.009	0.9989
		12-w T	0.339	0.0290	0.383	0.0000	0.283	0.0017	0.299	0.0020
PC(40:1)	12-w C	14-m C	n.d.	n.d.	n.d.	n.d.	0.225	0.1446	0.491	0.0012
		12-w T	n.d.	n.d.	n.d.	n.d.	0.133	0.5383	0.037	0.9796
		14-m T	n.d.	n.d.	n.d.	n.d.	0.469	0.0019	0.618	0.0001
	14-m C	12-w C	n.d.	n.d.	n.d.	n.d.	-0.225	0.1446	-0.491	0.0012
		12-w T	n.d.	n.d.	n.d.	n.d.	-0.093	0.7734	-0.454	0.0022
		14-m T	n.d.	n.d.	n.d.	n.d.	0.243	0.1071	0.127	0.5618
	12-w T	12-w C	n.d.	n.d.	n.d.	n.d.	-0.133	0.5383	-0.037	0.9796
		14-m C	n.d.	n.d.	n.d.	n.d.	0.093	0.7734	0.454	0.0022
		14-m T	n.d.	n.d.	n.d.	n.d.	0.336	0.0205	0.581	0.0003
	14-m T	12-w C	n.d.	n.d.	n.d.	n.d.	-0.469	0.0019	-0.618	0.0001
		14-m C	n.d.	n.d.	n.d.	n.d.	-0.243	0.1071	-0.127	0.5618
		12-w T	n.d.	n.d.	n.d.	n.d.	-0.336	0.0205	-0.581	0.0003
PC(42:1)	12-w C	14-m C	n.d.	n.d.	n.d.	n.d.	0.655	0.0220	0.996	0.0002
		12-w T	n.d.	n.d.	n.d.	n.d.	0.181	0.7798	0.072	0.9681
		14-m T	n.d.	n.d.	n.d.	n.d.	0.962	0.0014	1.232	0.0000
	14-m C	12-w C	n.d.	n.d.	n.d.	n.d.	-0.655	0.0220	-0.996	0.0002
		12-w T	n.d.	n.d.	n.d.	n.d.	-0.474	0.1123	-0.924	0.0005
		14-m T	n.d.	n.d.	n.d.	n.d.	0.307	0.4086	0.236	0.4842
	12-w T	12-w C	n.d.	n.d.	n.d.	n.d.	-0.181	0.7798	-0.072	0.9681
		14-m C	n.d.	n.d.	n.d.	n.d.	0.474	0.1123	0.924	0.0005
		14-m T	n.d.	n.d.	n.d.	n.d.	0.781	0.0069	1.160	0.0001
	14-m T	12-w C	n.d.	n.d.	n.d.	n.d.	-0.962	0.0014	-1.232	0.0000
		14-m C	n.d.	n.d.	n.d.	n.d.	-0.307	0.4086	-0.236	0.4842
		12-w T	n.d.	n.d.	n.d.	n.d.	-0.781	0.0069	-1.160	0.0001

Acetyl-L-carnitine/L-carnitine	12-w C	14-m C	-0.186	0.2439	-0.118	0.7519	0.028	0.9707	-0.094	0.5004
		12-w T	0.241	0.0949	0.174	0.4779	0.199	0.0402	0.035	0.9510
		14-m T	0.186	0.2411	0.279	0.1348	0.322	0.0015	0.149	0.1591
	14-m C	12-w C	0.186	0.2439	0.118	0.7519	-0.028	0.9707	0.094	0.5004
		12-w T	0.427	0.0030	0.292	0.1139	0.171	0.0851	0.129	0.2533
		14-m T	0.372	0.0084	0.397	0.0245	0.294	0.0031	0.244	0.0136
	12-w T	12-w C	-0.241	0.0949	-0.174	0.4779	-0.199	0.0402	-0.035	0.9510
		14-m C	-0.427	0.0030	-0.292	0.1139	-0.171	0.0851	-0.129	0.2533
		14-m T	-0.055	0.9327	0.105	0.8066	0.123	0.2743	0.115	0.3435
	14-m T	12-w C	-0.186	0.2411	-0.279	0.1348	-0.322	0.0015	-0.149	0.1591
		14-m C	-0.372	0.0084	-0.397	0.0245	-0.294	0.0031	-0.244	0.0136
		12-w T	0.055	0.9327	-0.105	0.8066	-0.123	0.2743	-0.115	0.3435
ACh/Acetyl-L-carnitine	12-w C	14-m C	0.302	0.0501	0.273	0.1398	n.d.	n.d.	n.d.	n.d.
		12-w T	-0.461	0.0033	-0.444	0.0110	n.d.	n.d.	n.d.	n.d.
		14-m T	-0.097	0.7781	-0.129	0.6870	n.d.	n.d.	n.d.	n.d.
	14-m C	12-w C	-0.302	0.0501	-0.273	0.1398	n.d.	n.d.	n.d.	n.d.
		12-w T	-0.763	0.0000	-0.717	0.0002	n.d.	n.d.	n.d.	n.d.
		14-m T	-0.399	0.0095	-0.402	0.0208	n.d.	n.d.	n.d.	n.d.
	12-w T	12-w C	0.461	0.0033	0.444	0.0110	n.d.	n.d.	n.d.	n.d.
		14-m C	0.763	0.0000	0.717	0.0002	n.d.	n.d.	n.d.	n.d.
		14-m T	0.364	0.0173	0.315	0.0766	n.d.	n.d.	n.d.	n.d.
	14-m T	12-w C	0.097	0.7781	0.129	0.6870	n.d.	n.d.	n.d.	n.d.
		14-m C	0.399	0.0095	0.402	0.0208	n.d.	n.d.	n.d.	n.d.
		12-w T	-0.364	0.0173	-0.315	0.0766	n.d.	n.d.	n.d.	n.d.
alpha-tocopherol	12-w C	14-m C	-0.177	0.0488	-0.093	0.5512	n.d.	n.d.	n.d.	n.d.
		12-w T	0.063	0.7155	0.023	0.9872	n.d.	n.d.	n.d.	n.d.
		14-m T	-0.170	0.0600	-0.087	0.6018	n.d.	n.d.	n.d.	n.d.
	14-m C	12-w C	0.177	0.0488	0.093	0.5512	n.d.	n.d.	n.d.	n.d.
		12-w T	0.240	0.0076	0.115	0.3756	n.d.	n.d.	n.d.	n.d.
		14-m T	0.007	0.9993	0.006	0.9997	n.d.	n.d.	n.d.	n.d.
	12-w T	12-w C	-0.063	0.7155	-0.023	0.9872	n.d.	n.d.	n.d.	n.d.
		14-m C	-0.240	0.0076	-0.115	0.3756	n.d.	n.d.	n.d.	n.d.
		14-m T	-0.233	0.0093	-0.109	0.4191	n.d.	n.d.	n.d.	n.d.
	14-m T	12-w C	0.170	0.0600	0.087	0.6018	n.d.	n.d.	n.d.	n.d.
		14-m C	-0.007	0.9993	-0.006	0.9997	n.d.	n.d.	n.d.	n.d.
		12-w T	0.233	0.0093	0.109	0.4191	n.d.	n.d.	n.d.	n.d.

*Mean difference of the compared groups, i.e., mean value of log transformed metabolite intensity levels in the group at column A minus mean value of log transformed metabolite intensity levels in the group at column B, at significance level α=0.05. Cx and cc were not included since more specific areas, i.e., RS, mfb, were considered as adequate statistical representatives. Abbreviations: Hip, hippocampus; RS, retropsplenial cortex; mfb, medial forebrain bundle; Str, striatum; n.c., not computable; n.d., not determined.

Table S4. PLS-DA model validation with permutation tests.

Permutation tests (100 permutations) were applied through randomly re-ordering the response variables and the newly derived R^2 and Q^2 were plotted against the degree of correlation between the permuted and original data. Models derived after permutation showed considerably inferior statistics ($R^2_{\text{intercept}}$ <<0.5, $Q^2_{\text{intercept}}$ <0.0) indicating the robustness of the original models.

Model (based on the brain structure)	$R^2_{\text{intercept}}$ (average of all cleasses)	$Q^2_{\text{intercept}}$ (average of all classes)
cc	0.329	-0.265
Cx	0.251	-0.291
mfb	0.152	-0.255
Str	0.133	-0.241
Hip/ RS age	0.189	-0.294
Hip/RS treatment	0.108	-0.286

Abbreviations: cc, corpus callosum; Cx, cortex; mfb, medial forebrain bundle; Hip, hippocampus, RS, retrosplenial cortex.

References

(1) Worley, B.; Powers, R. PCA as a practical indicator of OPLS-DA model reliability. *Curr Metabolomics* **2016**, *4*, 97-103.

(2) van der Hooft, J. J.; Ridder, L.; Barrett, M. P.; Burgess, K. E. Enhanced acylcarnitine annotation in high-resolution mass spectrometry data: fragmentation analysis for the classification and annotation of acylcarnitines. *Front Bioeng Biotechnol* **2015**, *3*, 26.

(3) Shariatgorji, M.; Nilsson, A.; Fridjonsdottir, E.; Vallianatou, T.; Kallback, P.; Katan, L.; Savmarker, J.; Mantas, I.; Zhang, X.; Bezard, E.; Svenningsson, P.; Odell, L. R.; Andren, P. E. Comprehensive mapping of neurotransmitter networks by MALDI-MS imaging. *Nat Methods* **2019**, *16*, 1021-1028.

(4) Paxinos, G.; Franklin, K. B. J. *Paxinos and Franklin's the mouse brain in stereotaxic coordinates*, 4th ed.; Elsevier Academic Press: Amsterdam, 2013.
